# Snapshots during the catalytic cycle of a histidine acid phytase reveal an induced-fit structural mechanism

**DOI:** 10.1074/jbc.RA120.015925

**Published:** 2020-10-14

**Authors:** Isabella M. Acquistapace, Monika A. Zi¸etek, Arthur W. H. Li, Melissa Salmon, Imke Kühn, Mike R. Bedford, Charles A. Brearley, Andrew M. Hemmings

**Affiliations:** 1 School of Biological Sciences, University of East Anglia, Norwich, United Kingdom; 2 AB Vista, Darmstadt, Germany; 3 AB Vista, Marlborough, Wiltshire, United Kingdom; 4 School of Chemistry, University of East Anglia, Norwich, United Kingdom

**Keywords:** phytase, cell surface protein, induced fit, enzyme mechanism, enzyme structure, crystallography, thermostabilization, stereospecificity, inositol phosphate, cell surface enzyme, enzyme mechanism, substrate specificity, enzyme mutation, structural biology, structure-function, induced fit, phytase, phytic acid, thermostabilization

## Abstract

Highly engineered phytases, which sequentially hydrolyze the hexakisphosphate ester of inositol known as phytic acid, are routinely added to the feeds of monogastric animals to improve phosphate bioavailability. New phytases are sought as starting points to further optimize the rate and extent of dephosphorylation of phytate in the animal digestive tract. Multiple inositol polyphosphate phosphatases (MINPPs) are clade 2 histidine phosphatases (HP2P) able to carry out the stepwise hydrolysis of phytate. MINPPs are not restricted by a strong positional specificity making them attractive targets for development as feed enzymes. Here, we describe the characterization of a MINPP from the Gram-positive bacterium *Bifidobacterium longum* (*Bl*MINPP). *Bl*MINPP has a typical HP2P-fold but, unusually, possesses a large α-domain polypeptide insertion relative to other MINPPs. This insertion, termed the U-loop, spans the active site and contributes to substrate specificity pockets underpopulated in other HP2Ps. Mutagenesis of U-loop residues reveals its contribution to enzyme kinetics and thermostability. Moreover, four crystal structures of the protein along the catalytic cycle capture, for the first time in an HP2P, a large ligand-driven α-domain motion essential to allow substrate access to the active site. This motion recruits residues both downstream of a molecular hinge and on the U-loop to participate in specificity subsites, and mutagenesis identified a mobile lysine residue as a key determinant of positional specificity of the enzyme. Taken together, these data provide important new insights to the factors determining stability, substrate recognition, and the structural mechanism of hydrolysis in this industrially important group of enzymes.

Phytic acid (*myo-*inositol hexakisphosphate; InsP_6_) (Fig. S1) is the major storage form of phosphorous (50–80% of total P) in grains, oil seeds, and beans used in common animal feeds (1). Phytases are the phosphomonoesterases that catalyze the sequential dephosphorylation of phytate necessary to release this phosphorous in its utilizable form, orthophosphate. Animals rely on phytases produced by their commensal microbiota. However, in monogastrics, such as pigs and poultry, the capacity of endogeneous phytases to break down dietary phytic acid is limited. Passage of undigested phytic acid to the environment in areas of intensive animal husbandry can lead to the formation of algal blooms in aquatic ecosystems from nutrient overloading. This in turn leads to eutrophication, which has been shown to reduce benthic biomass and biodiversity (2).

**Figure 1.**
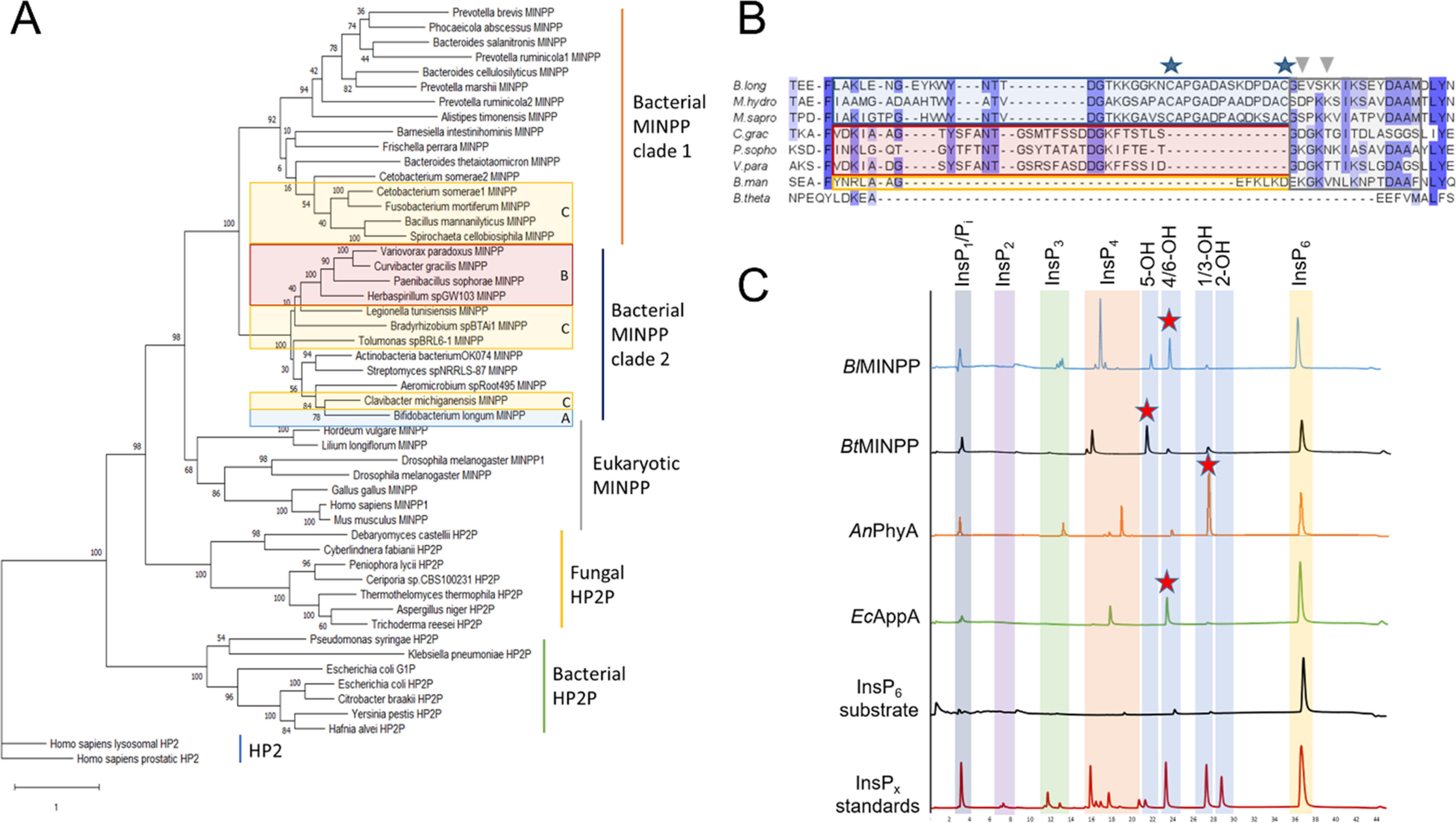
*A,* maximum likelihood phylogenetic tree resulting from the alignment of 51 clade 2 histidine phosphatases (Pfam family: his_phos_2). The sequences correspond mainly to phytases with the exception of two histidine phosphatases without phytase activity (HP2, *light blue*) on which the tree is rooted. Different branches were identified as representatives of family subgroups: bacterial phytases (*green*), fungal phytases (*yellow*), eukaryotic MINPPs (*gray*), and two clades of bacterial MINPPs: clade 1 (*blue*) and clade 2 (*orange*). Sequences were inspected for the presence of a U-loop insertion and highlighted as follows: *blue boxes,* sequences contain a type A U-loop; *red boxes*, contain a type B U-loop; *yellow boxes*, contain a type C U-loop. *B,* sequence alignment of representative U-loop-containing MINPPs. All three U-loop types contain a conserved region, highlighted in a *gray shaded box*, which includes the residues potentially involved in contacts with substrate. The remaining regions are less conserved and can be either long (type A U-loop, *blue box*, characterized by an insertion between two cysteines potentially forming a disulfide bridge), medium (type B U-loop, *red box*), or short (type C U-loop, *yellow box*). Most sequences containing a U-loop contain a “DAAM” motif downstream of the insertion. U-loops are found in bacterial MINPPs from either clade A or B. *Stars* highlight positions of cysteine residues conserved in type A U-loops, whereas *inverted triangles* point to residues contributing to phytate specificity pockets. *C,* HPLC chromatograms of the products of InsP_6_ hydrolysis by *Bl*MINPP, *Bt*MINPP, *A. niger* PhyA (*An*PhyA), and *E. coli* AppA (*Ec*AppA). *x* axis, retention time (min); *y* axis, intensity of UV absorbance at 290 nm. Chromatograms are labeled by enzyme. The major InsP_5_ product for each enzyme is indicated by a *red star*. Chromatograms of the undigested substrate (InsP_6_ substrate) and an acid hydrolysate of the substrate (InsP*_x_* standards) are shown for reference. The elution volume ranges for the various inositol polyphosphate products are highlighted by *vertical colored backgrounds* (note that the notation for the InsP_5_ products is based on the identity of the free hydroxyl group of the intermediate).

To increase the efficiency of conversion of phytic acid into available dietary phosphate, animal feeds are routinely supplemented with exogeneous phytases. Of the major classes of phytase, those belonging to clade 2 of the histidine phosphatase superfamily (Pfam code: PF00328, His_Phos_2; HP2) have found most widespread use. This is due both to their high specific activity toward InsP_6_ and to possessing pH optima in the acid regime. Notable examples are the periplasmic phytase, AppA, from *Escherichia coli*, and the secreted phytases produced by *Aspergilli* such as PhyA of *Aspergillus niger* (3, 4). This area is the subject of ongoing interest in attempts to uncover both new enzymes and engineered variants with high activity, tailored specificity and enhanced stability to environmental assault (5, 6); the latter frequently associated with the high temperatures to which these enzymes are exposed during the feed pelleting process.

HP2 phytases (HP2P) can be grouped according to the specific position of the phosphate ester group of the substrate at which hydrolysis is initiated, *e.g.* as 1D-3-phytases (EC 3.1.3.8) or 1D-4-phytases (EC 3.1.3.26). The EC 3.1.3.26 signifier refers to seminal characterization of the enantiomerism of products generated by the action of wheat bran phytase (reviewed in Ref. 7). 1D-6-phytases, exemplified by *E. coli* AppA, are acid phosphatases (EC 3.1.3.2) and act via an obligatory phosphohistidine intermediate (Fig. S2) (8). The crystal structures of a variety of HP2Ps have been solved (Table S1) from a variety of both bacterial (9–13) and fungal (14–16) sources. The active site cleft of these lies between two structural domains: an α- and an α/β-domain. The fold of the latter is well-conserved in HP2P, whereas the α-domain is subject to enzyme-specific changes (17). At the base of this active site cleft are found two amino acid sequence motifs, the first possesses a consensus RHG*X*R*X*h sequence motif (where h represents a hydrophobic amino acid and *X* is any amino acid) involved in substrate binding and containing the eponymous nucleophilic histidine observed in all HP2 family members (8, 18–20). The catalytic proton donor is found in a second, short, conserved HD sequence motif positioned such that the aspartic acid residue is the consensus proton donor in the catalytic mechanism (9, 20–23). The proton donor is required for the release of the lower inositol phosphorylated product of hydrolysis and orthophosphate with regeneration of the catalytic histidine.

**Figure 2.**
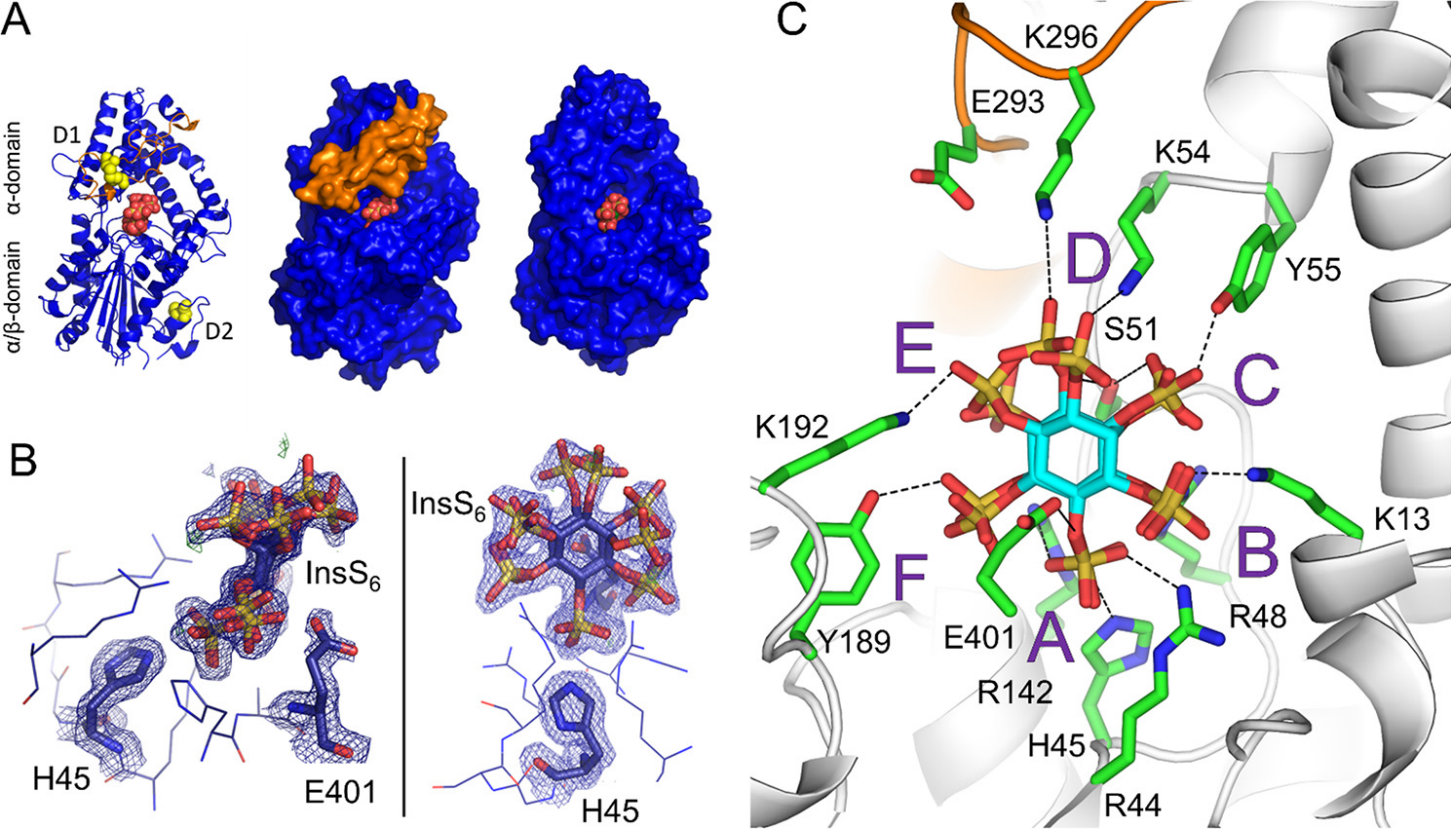
**The crystal structure of the complex of *Bl*MINPP with the substrate analog inhibitor, InsS_6_.** *A,* the U-loop obscures access of inhibitor to active center. *Left panel*: a cartoon representation of the fold of *Bl*MINPP shown in *blue* except for the residues of the U-loop (257-300), which are colored *orange*. Atoms of the inhibitor bound between the lower α/β-domain and the upper α-domain are shown in *sphere format* and colored *orange* (sulfur) and *red* (oxygen). The four cysteine residues forming two disulfide bridges (labeled *D1* and *D2*) are shown as *spheres* and colored *yellow*. *Center panel*, a molecular surface representation of the *Bl*MINPP structure. Orientation and coloring as in *left panel*. *Right panel,* molecular surface representation of the structure of *Bt*MINPP in complex with InsS_6_ (PDB 4FDU). Orientation and coloring are as described in the *left panel*. *B,* orthogonal views of the electron density for selected active site residues and the bound inhibitor molecule. Inhibitor, two conformations. Residues His-45 and Glu-401 are shown in stick format. Other residues of the two active site signature sequence motifs RHG*X*R (residues 44-48) and HAE (residues 399-401), along with Arg-142 are shown in wire format. *Dark blue hatching* shows the difference Fourier (2m*F_o_* – D*F_c_*) electron density map contoured at 1.0σ. *C,* closeup view of bound inhibitor and definition of specificity subsite nomenclature. Enzyme shown in cartoon format and colored *gray,* except for the residues of the U-loop (257-300), which are colored *orange*. Bound InsS_6_ (two conformations) are shown with carbon colored *cyan*, active site residues with carbon colored *green* and labeled. Polar interactions are indicated by *dashed lines*. Specificity subsites are labeled *A–F* such that the analog of the scissile phosphate occupies subsite A and the remaining subsites are arrayed in a counterclockwise sense when observed from the viewpoint adopted in this figure.

**Table 1:** **X-ray data collection and structure refinement statistics** Numbers in parentheses refer to high resolution data.

Protein	Apo	InsS_6_ complex	Phosphohistidine intermediate	P_i_ complex
**PDB ID**	6RXD	6RXE	6RXF	6RXG
**Data collection**				
Beamline	Diamond I02	Diamond I02	Diamond I03	Diamond I03
Wavelength (Å)	0.9795	0.9795	0.9763	0.9762
Space group	P1	P2_1_	P1	P1
Cell parameters				
*a*, *b*, *c* (Å)	54.7, 72.9, 88.2	70.8, 106.4, 76.5	55.5, 73.1, 89.0	54.6, 72.8, 87.3
α, β, γ (°)	71.6, 72.1, 77.2	90.0, 112.6, 90.0	71.3, 73.0, 79.0	71.4, 72.2, 76.8
Resolution limit (Å)	46.53-1.65 (1.69-1.65)	70.57-1.84 (1.91-1.84)	68.9-2.40 (2.44-2.40)	68.23-1.71 (1.75–1.71)
*R* merge	0.059 (0.45)	0.159 (0.625)	0.233 (0.826)	0.063 (0.525)
CC1/2	0.994 (0.645)	0.979 (0.603)	0.925 (0.328)	0.984 (0.503)
〈(*I*)/σ(*I*)〉	8.4 (1.9)	4.6 (1.6)	2.3 (1.5)	7.5 (1.4)
Completeness (%)	96.3 (94.9)	99.65 (98.9)	94.6 (88.8)	95.5 (95.0)
Multiplicity	2.2 (2.1)	3.4 (3.3)	3.4 (3.3)	1.9 (1.9)
Overall temperature factor (Å^2^)	18.6	10.9	25.5	17.3
**Refinement**				
Protein monomers per asymmetric unit	2	2	2	2
Number of protein residues	509	509	509	509
Total atoms	9,328	9,374	8,117	15,951
Water molecules	1,287	1,447	286	805
*R*_work_	15.3%	20.9%	18.5%	14.7%
*R*_free_	17.7%	23.8%	23.9%	19.4%
Ramachandran analysis (%)				
Most favored	98.11%	98.1%	96.8%	97.7%
Outliers	0.28%	0.2%	0.2%	0.3%
RMS deviations				
Bonds (Å)	0.006	0.004	0.006	0.009
Angles (°)	1.008	0.70	0.80	0.927
Planes (Å)	0.004	0.004	0.005	0.006
Mean atomic *B*-value (Å2)	23.0	16.99	28.56	27.9
Macromolecule	NA*^a^*	15.16	28.31	27.3
Ligands	NA	15.85	45.06	
Solvent	26.95	26.95	34.77	38.8

*^a^* NA, not applicable.

The multiple inositol polyphosphate phosphatases (MINPPs) constitute a distinct evolutionary group within clade 2 of the histidine phosphatase superfamily (21, 24). Examples are found in *Bacteria* and *Eukarya* but MINPPs have not yet been identified in the domain *Archaea* (25). Like other HP2P enzymes, MINPPs carry the RHG*X*R*X*h sequence motif involved in substrate binding and catalysis. However, instead of an HD proton donor motif, the residues of an amino acid triplet, frequently with the sequence HAE, are presumed to provide an equivalent function (22). MINPPs were so-named because of their broad substrate specificity when compared with other HP2P. This allows, for example, the removal of the 3-phosphate of 2,3-bisphosphoglycerate, by so doing expanding the regulatory capacity of the Rapoport–Luebering glycolytic shunt in *Dictyostelium*, birds, and mammals (26). In the same vein, unlike HD-motif containing HP2P enzymes, MINPPs lack a strong initial positional hydrolytic specificity toward phytic acid, producing a variety of InsP_5_s and lower InsP*_x_* (22, 27, 28). As with the eukaryotic enzymes, bacterial MINPPs also lack a strong initial positional specificity, generating in the process a variety of InsP_5_s and lower inositol polyphosphates. These partially dephosphorylated intermediates may have a variety of functions. For example, an extracellular MINPP released in outer membrane vesicles by the major Gram-negative human gut symbiont, *Bacteroides thetaiotaomicron* (22) has been suggested to participate in cross-kingdom cell-to-cell signaling by promoting intracellular Ca^2+^ signaling in intestinal epithelial cells. Despite this, the function of many MINPPs is still uncertain despite evidence of a role of these enzymes in a variety of cellular processes and organisms (29–32).

Extracellular enzymes generally show high thermostability (33) and so, based on the premise that bacterial extracellular MINPPs may represent a useful source of enzymes as next-generation animal feed phytases, we carried out an analysis of their amino acid sequences. In the process, we identified a subset bearing large α-domain polypeptide insertions (termed U-loops). To gain a wider understanding of the implications of these insertions on the properties of these enzymes, we carried out a crystal structure and mutagenic analysis of a representative member, *Bl*MINPP, a moderately thermophilic, membrane-anchored extracellular enzyme. Interestingly, whereas the presence of an intra-U-loop disulfide bridge increased overall protein thermostability by 10 °C, it was found that residues of the loop unexpectedly also contributed to substrate specificity. Furthermore, four crystal structures were determined providing snapshots along the catalytic cycle of the enzyme revealing a large, ligand-driven domain motion previously unseen in the HP2P family. These results suggest evolution of polypeptide insertions may present a route for enhanced thermostability of extracellular phytases, but their presence imposes additional requirements for enhanced molecular flexibility necessary for catalysis to occur.

## Results

### The U-loop: a polypeptide insertion in extracellular MINPPs

Phylogenetic analysis of HP2Ps reveals the expected separation of MINPP and nonMINPP sequences (Fig. 1*A*). The nonMINPPs can be identified as those from fungal (eHP2P) or bacterial (bHP2P) sources. The MINPPs can be similarly divided into those from *Eukaryota* or *Bacteria*. The bacterial MINPPs are further divided in two clades, named here clades 1 and clade 2. Three groups of polypeptide inserts in MINPP sequences were detected (we refer to these as U-loops) and given the identifiers A, B, or C based on insert length. The sequences of the three loop types do not align well with each other, save for a conserved region toward the C-terminal end (Figs. 1*B*, Fig. S3). This region is followed by a characteristic tetrapeptide DAAM motif, which is absent in sequences that do not contain a U-loop. The type A U-loop is the longest, containing two conserved cysteine residues. It is found in clade 2 MINPPs as extracellular membrane-anchored enzymes of Gram-positive bacteria. Of these, *Bifidobacterium longum* is a major human gut bacterium present from infancy through to adult life (34). Type B is a medium length loop also found in clade 2 MINPPs. It can be viewed as resulting from a deletion of the amino acids between the two conserved cysteine residues of the type A insert (Fig. 1*B*). Enzymes containing this loop are mostly predicted to be membrane-anchored lipoproteins, characterized by a SEC/SPII signal peptide. Type C is a short U-loop found in both clade 1 and 2 MINPPs. The majority of enzymes with type C inserts appear to be either lipoproteins (possessing a SEC/SPII signal peptide) or have a signal peptide (SEC/SPI N-terminal signal peptide).

**Figure 3.**
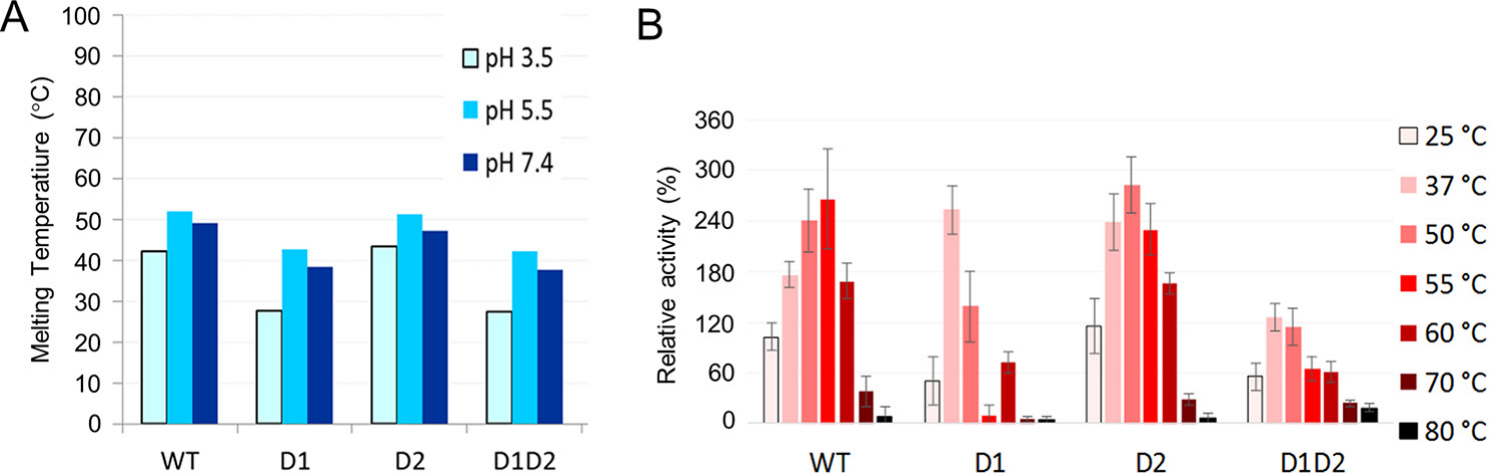
**The role of the U-loop in determining enzyme stability.** *A,* melting temperatures of *Bl*MINPP disulfide bridge knock-out mutants. Key to substitutions: *D1,* C278A/C291A; *D2,* C483A/C501; *D1D2,* C278A/C291A/C483A/C501. Results of differential scanning calorimetry at pH 3.5, 5.5, and 7.4 of *Bl*MINPP WT (*WT*) and mutants D1, D2, and D1D2 are shown. *y* axis, melting temperature (°C); *x* axis, protein samples. Reactions were carried after incubation for 30 min at 25, 37, 50, 55, 60, 70, and 80 °C. Mutants D1 and D1D2 shows a 10 °C lower melting temperature when compared with the WT enzyme. Due to DSC sample requirements only a single measurement was made at each pH. As such, these results should be considered tentative. *B,* recovery after heating assays of *Bl*MINPP disulfide bridge knock-out mutants. The enzyme was incubated at pH 5.5 and residual phytase activity determined after cooling to room temperature. Activity expressed as a percentage (%) relative to that recovered by of the WT enzyme after heating to 25 °C.

The extracellular type A U-loop-containing MINPP from *B. longum* subsp. *infantis* ATCC 15697 (*Bl*MINPP) is reported to be relatively thermostable, preserving 44% of activity after incubation at 80 °C for 15 min (35). It has a sequence identity of only 23% compared with the mesophilic, nonU-loop-containing MINPP secreted in lipid vesicles from the previously characterized Gram-negative human gut bacterium *B. thetaiotaomicron* (*Bt*MINPP) (22). It also resides in a different clade. It is larger than the *Bacteroides* enzyme, bearing nearly 100 additional amino acids in multiple polypeptide insertions and, based on the known crystal structure of the latter, at least one of these was presumed to face the active site cleft. For these reasons we decided to probe the structure-function basis for the elevated thermostability of this type A U-loop enzyme.

### BlMINPP displays typical MINPP catalytic positional specificity

The N-terminal signal peptide and a C-terminal sortase-dependent cell wall-anchoring L(P/A)*X*TG domain were removed from the cloned *Bl*MINPP construct, and the enzyme expressed intracellularly in *E. coli* and purified. The recombinant enzyme has a pH profile of activity toward phytate, which is typical of HP2Ps, displaying a maximum activity at pH 5.5 (35). HPLC separation of hydrolysis products following digestion of InsP_6_ reveals the lower positional specificity toward this substrate that is characteristic of MINPPs (Fig. 1*C*) relative to other HP2Ps. In this way, the major InsP_5_ observed is 1D- and/or 1L-Ins(1,2,3,5,6)P_5_, hereafter [4/6-OH]InsP_5_ (note that the enantiomers are not resolvable). The *meso*-compound Ins(1,2,3,4,6)P_5_, hereafter [5-OH]InP_5_, is also produced but at a lower level (the ratio of these products is roughly 2:1). A much smaller amount of 1D- and/or 1L-Ins(1,2,4,5,6)P_5_, hereafter [1/3-OH]InsP_5_, is also observed. This differs subtly from *Bt*MINPP which produces [5-OH]InsP_5_ as its major product (22). Hydrolytic activity also appears to stop with production of InsP_3_s (35), differing again from the *Bacteroides* enzyme that produces lower inositol mono/polyphosphates. (Although InsP_2_s are not observed in chromatograms separating the products of action of BlMINPP, it is important to note that InsP_1_ and P_i_ are not resolvable using our HPLC method and so hydrolysis beyond InsP_3_ intermediates may be occurring.) This behavior contrasts with that of other bHP2Ps such as *E.coli* AppA, which is accepted to possess high positional specificity, generating a majority 1D-Ins(1,2,3,4,5)P_5_ product, hereafter [6-OH]InsP_5_ with a small amount of 1D-Ins(1,2,4,5)P_5_, [1/3-OH]InsP_5_ (Fig. 1*C*) and a well-characterized 6/1/3/4/5 dephosphorylation pathway, ultimately yielding Ins2P (36). In an attempt to cast light on the structural basis for the differing positional specificities of *Bl*MINPP and *Bt*MINPP we embarked on an X-ray crystal structure determination of the *B. longum* enzyme.

### Phytate specificity subsites from the crystal structure of the BlMINPP-InsS_6_ complex

A crystal of the purified recombinant enzyme grown in the presence of an excess InsS_6_, a nonhydrolyzable phytate mimic, diffracted to 1.84 Å resolution. These data were used solve the X-ray crystal structure of the complex by molecular replacement with the InsS_6_-bound structure of *Bt*MINPP employed as search model. Despite low sequence identity, the overall folds of the two MINPPs are very similar. In keeping with previously reported crystal structures of HP2P family members, the structure of *Bl*MINPP consists of two domains, an α/β-domain and an α-domain (Fig. 2*A*). For both *Bl*MINPP and *Bt*MINPP the α-domain resembles that seen in the crystal structures eHP2Ps from the closely related fungi *Aspergillus niger* (14) and *Aspergillus fumigatus* (37). The catalytic center of *Bl*MINPP consists of the nucleophilic histidine (His-45), three arginine residues (Arg-44, Arg-48, and Arg-142), which coordinate the scissile phosphate during catalysis, and the amino acid triplet HAE (residues 339-401) where the glutamic acid residue acts as the presumed proton donor during catalysis (22). Glutamic acid here replaces the aspartic acid seen in a corresponding HD sequence motif commonly found in nonMINPP HP2P family members (20). The inhibitor binds in the active site cleft in a manner presumed to mimic that of phytic acid. However, inspection of a molecular surface representation of the complex suggests that egress of the ligand is obstructed (Fig. 2*A*). This obstruction would presumably also hinder diffusion of partially dephosphorylated inositol polyphosphate hydrolysis products. This is in contrast to the situation observed with *Bt*MINPP (Fig. 2*A*) and with all bHP2P for which corresponding crystal structures are available.

The structure of the *Bl*MINPP-InsS_6_ complex reveals static disorder in the inhibitor, which is bound in two orientations (Fig. 2*B*, Fig. S4). The first of these has the sulfate group at position 1D-4 of the inositol ring positioned proximal to the nucleophilic histidine. The other has the 1D-6 sulfate in this orientation. The pairs of inhibitor molecules bound to the two protein monomers in the crystallographic asymmetric unit have occupancy ratios 0.63:0.37 and 0.53:0.47 (S4:S6) suggesting little intrinsic preference between the two orientations of binding. Although noting that [4-OH]1D-InsP_5_ and [6-OH]1D-InsP_5_ = [4-OH]1L-InsP_5_, enantiomers cannot be separated by our HPLC approach, our observation of disorder in binding of a substrate-mimic may reflect a dual 1D-4/6-positional specificity for the enzyme. Certainly, the presence of conformational disorder (*i.e.* multiple bound conformations) in the InsS_6_ ligand is consistent with the lower positional stereospecificity observed for this enzyme relative to that seen for the more specific canonical HP2P (36, 38–40).

**Figure 4.**
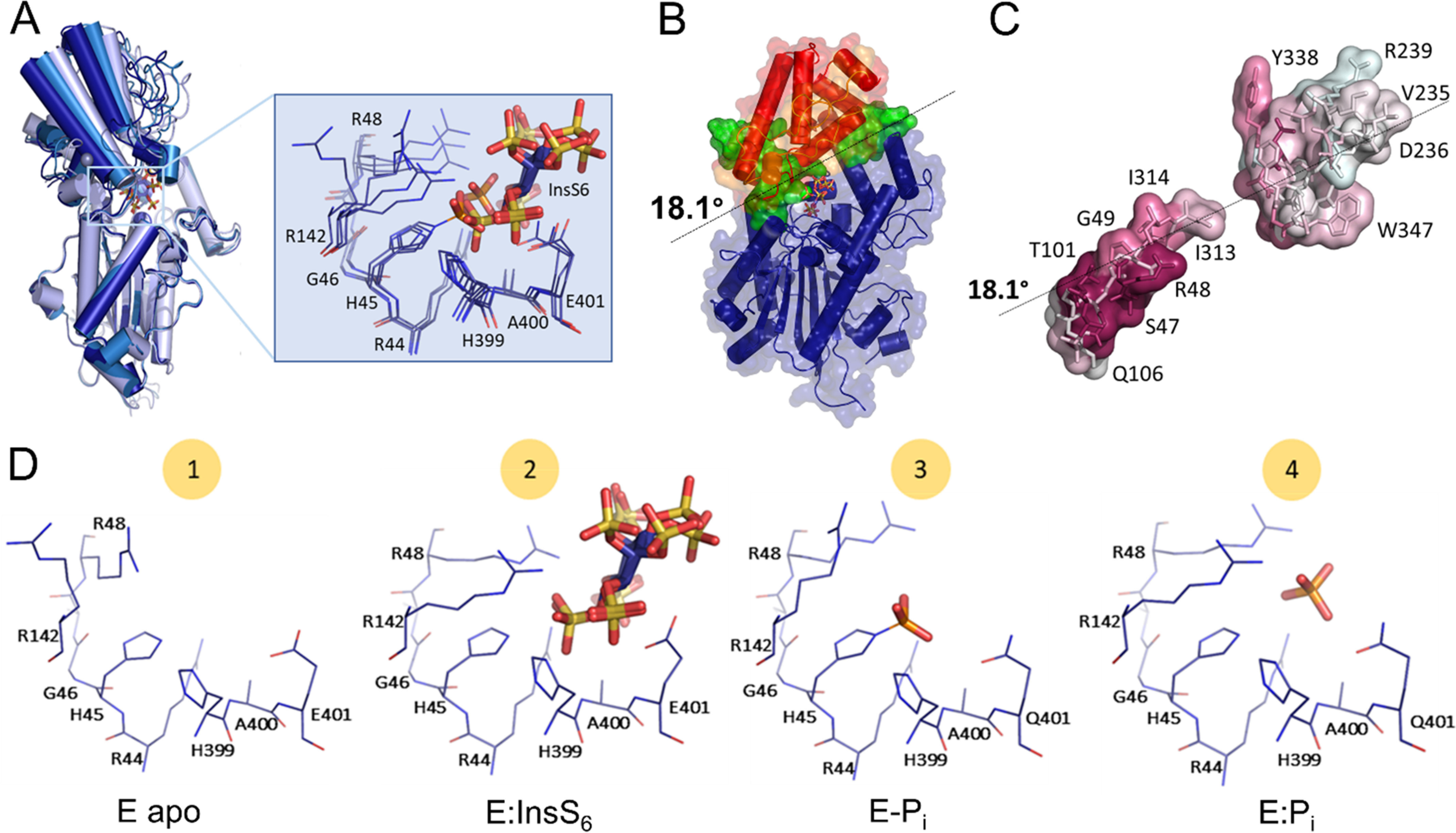
**Ligand-driven domain movement along the structural catalytic cycle of *Bl*MINPP.** *A,* superposition of the four *Bl*MINPP structures solved in this study shown in cartoon format: *blue,* apo-enzyme and phosphohistidine intermediate; *sky-blue,* phosphate-bound form; *light blue*, InsS_6_-bound form. α/β- and α-domains are indicated. A closeup of the catalytic core residues and inhibitor is also shown. Amino acids in conserved sequence motifs are labeled. InsS_6_, P_i_, and the phosphate group of phosphohistidine are represented in stick format. Large positional shifts are detected for Arg-48, Arg-142, and Glu-401 upon ligand binding. *B, Bl*MINPP regions involved in the domain movement (the lid). Areas highlighted: *blue,* fixed domain; *red* and *orange*, moving domain; *green*, hinge residues. The U-loop is *orange*-colored. The hinge rotational axis (18.1° from the apo-form in the structure above) is represented by *dotted lines*. *C,* hinge residues colored according to conservation as determined using ConSurf (45) according to a multisequence alignment of MINPP representative sequences. Darker surface colors indicate higher residue conservation. Residues most conserved are the pairs Ser-47, Arg-48, and Thr-101, Leu-100. The latter residue pair is part of a conserved “G*X*LT*X*_2_G” sequence motif. *D,* four snapshots along the catalytic cycle of *Bl*MINPP active site: 1) *E apo*: apo-protein; 2) *E:InsS_6_*: a model for the substrate-bound complex (*Bl*MINPP in complex with InsS_6_); 3) *E-Pi*: a catalytic intermediate (*Bl*MINPP E401Q-phosphohistidine intermediate); and 4) *E:Pi*: the product complex (*Bl*MINPP in complex with P_i_). Residues of the two active site signature sequence motifs RHG*X*R (residues 44-48) and HAE (residues 399-401) along with Arg-142 are shown in wire format.

The structure also provides a model for enzyme-substrate interactions. Specificity pockets for the binding of the six phosphates of InsP_6_ can be inferred by identifying all amino acids within 6 Å of each sulfate of the ligand in the complexed structure (the result is essentially identical irrespective of the orientation of binding). In this scheme, the residues of pocket A bind the scissile phosphate group and represent the catalytic center. This pocket is symmetrical in structure and charge, presenting guanidino groups from two arginine residues (Arg-48 and Arg-142) on each side of the phosphate. From a vantage point positioned behind the inositol ring and looking through it toward the A-subsite and arginines 48 and 142, the remaining specificity subsites are then labeled B–F in a counterclockwise fashion, following the order of decreasing sulfate number attached to the *myo-*inositol ring (Fig. 2*C*). It is likely that these are representative of the subsites involved in recognition and binding of InsP_6_. Of note is the fact that specificity pockets C, D, and E are more highly populated by active site residues than the equivalent sites in *Bt*MINPP and other HP2P such as *Ec*AppA (Table S2). U-loop residues account for this difference in the case of specificity subsite D. Specificity subsites C and E are also more highly populated in *Bl*MINPP. However, this appears to be due more to the presence of bulkier subsite residues in *Bl*MINPP than to local topology changes.

*Bl*MINPP is significantly larger than *Bt*MINPP and the majority of the additional residues are present as random coil. The exception to this are the 44 amino acids of the U-loop (residues 257 to 300) that lie on top of the active site, wrapped around the α-domain. This insertion is stabilized in position by interaction of U-loop residues residues Asn-266 and Asp-289 with the main chain amide and carbonyl groups, respectively, of Tyr-53. Tyr-53 lies only a few residues C-terminal to the consensus RHG*X*R*X*h active site sequence motif. Only three further polar interactions are found, these involving the side chains of Trp-264, Asn-277, and Asp-287, with the remainder of the interface predominantly hydrophobic in nature. No direct interactions are seen with active site residues. U-loop residues that close within 5 Å of the substrate analog are Glu-293 and Lys-296 (Fig. 2*C*) and form part of specificity subsite D. The U-loop thus helps define the active site, contributing to interactions in specificity subsite D and also partially obscuring product egress from it (Fig. 2*A*). These interactions are missing in the shorter, nonU-loop *Bacteroides* enzyme structure and simple sequence analysis suggests they are also absent in other important proteins in the family, such as the nonU-loop containing eMINPP proteins.

Thus, through its interactions with the bound substrate analog, the U-loop contributes to substrate recognition and, when compared with nonU-Loop enzymes, changes the shape and charge distribution of the active site cavity. The structure of *Bl*MINPP in complex with InsS_6_ also allows us to speculate on the likely roles of enzymes from the shorter U-loop classes. Inserts of types B and C are of sufficient length to provide equivalents to those residues from the leading strand of the hairpin that are able to interact with the substrate. Thus, the residues in *Bl*MINPP, which interact with the substrate analog in the D-specificity pocket, are predicted to be present in proteins from all U-loop classes and we would predict these loops to play analogous roles in determining substrate recognition and binding.

### The influence of the U-loop on protein stability and catalytic efficiency

Two cysteines on the U-loop, Cys-278 and Cys-291, form a disulfide bridge. A further disulfide (Cys-483–Cys-501) is observed in the α/β-domain, roughly 27 Å distant from the nucleophilic histidine and the active site (Fig. 2*A*). To test the roles of these disulfides on structure stabilization, the corresponding cysteine residue pairs were individually replaced by alanine mutagenesis and the resulting mutants assessed for stability by measurement of *T_m_* by differential scanning calorimetry, and by recovery of phytase activity after heating. Three disulfide deletion mutants were produced removing the Cys-278–Cys-291 disulfide (D1 mutant), the Cys-483–Cys-501 disulfide (D2 mutant), or both (D1D2 mutant). Differential scanning calorimetry revealed the melting temperatures of mutants D1 and D1D2 to be both reduced by ∼10 °C relative to the WT enzyme whereas that of mutant D2 was unchanged (Fig. 3*A*). MINPPs typically exhibit phytase activity maxima around pH 3.5, 5.5, and 7.5 (22). The depression in melting temperature observed for mutants D1 and D1D2 was effectively consistent across these pH values. These results suggest an involvement of the disulfide bridge Cys-278–Cys-291 and of the U-loop more generally in *Bl*MINPP structure stabilization. The recovery after heating of phytase activity of mutants D1 and D1D2 was also impaired (Fig. 3*B*). This enhanced sensitivity to heating following deletion of the U-loop disulfide may result from a general destabilization of the structure or simply from local melting of the U-loop structure leading to loss of stabilizing contacts of U-loop residues with substrate.

Only U-loop residues Glu-293 and Lys-296 approach within 5 Å of the bound substrate analog, the side chain of the former lying 4.6 Å from the sulfate in pocket D (Fig. 2*C*). We probed the roles of these residues by alanine mutagenesis (Fig. S5) (Table S3). *K_m_* was unperturbed for both mutants. *k*_cat_ was reduced for K296A, whereas for E293A it was almost 70% higher than the WT enzyme, suggesting that the U-loop contributes to fine tuning of catalytic activity, possibly by involvement with formation of the ES complex and/or product release.

**Figure 5.**
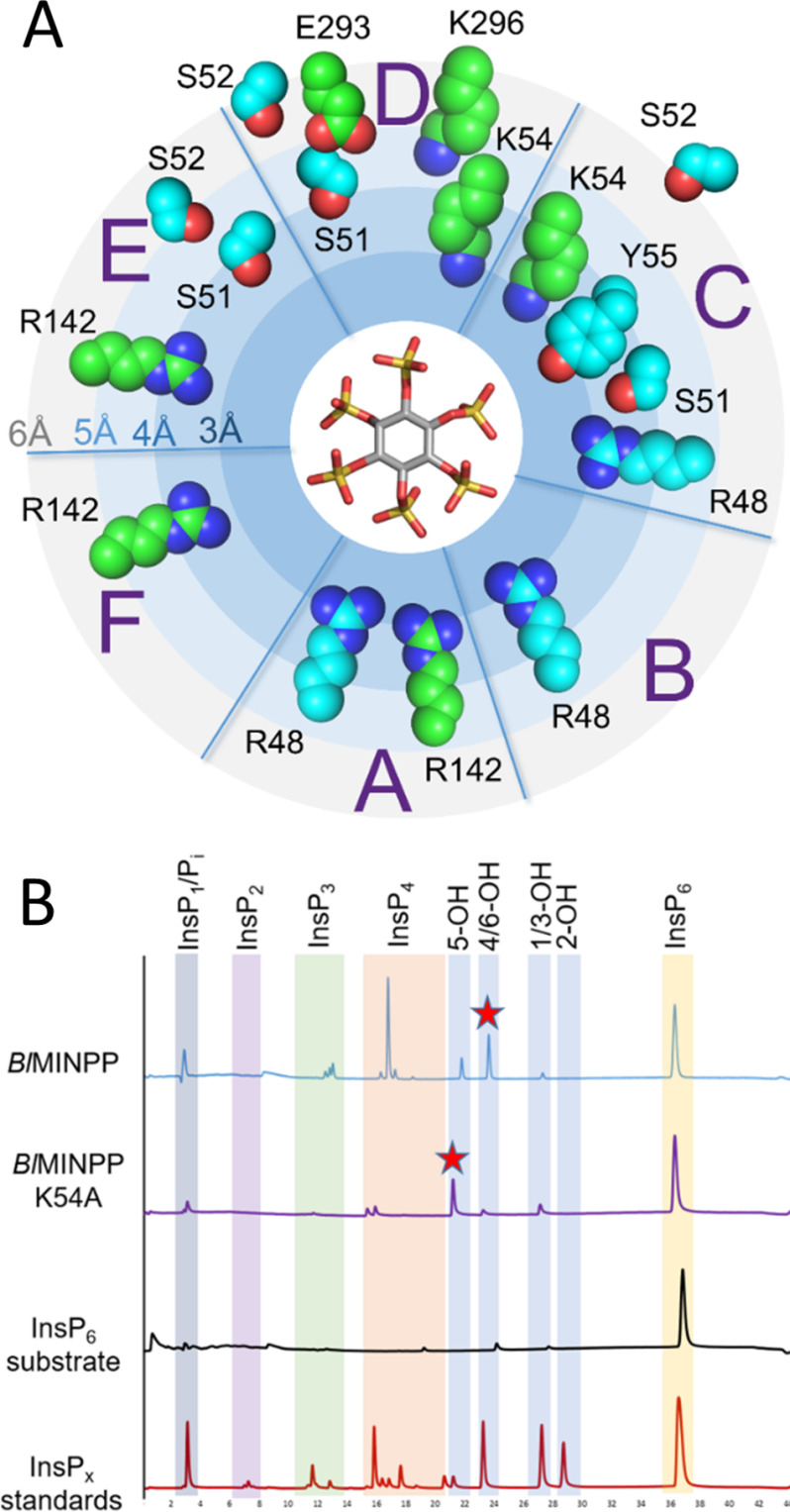
**Conformational change and its effect on the composition of specificity pockets and positional specificity of *Bl*MINPP.** *A,* a representation of the specificity subsites of *Bl*MINPP labeled *A–F* along with those active site residues that undergo signifcant movement (residues which move between 2 and 4 Å have carbon atoms colored *cyan* and those moving more than 4 Å on ligand binding are colored *green*). Residues that move less than 2 Å are 2 Å from their position in the apo-*Bl*MINPP structure to that found in the complex with InsS_6_. Residues shown in *sphere format* and *colored* so are not shown. The placement of the residues indicates the minimum polar contact distance with InsS_6_ (see Å scale on the *left-hand side*). In the center of the image sits a stick representation of InsS_6_ as a proxy for phytate positioned so that the sulfate attached to C6 is located in specificity pocket A. This orientation places the axial 2-sulfate in specificity pocket E. *B,* HPLC chromatograms of InsP_6_ hydrolysis by *Bl*MINPP WT and active site mutant K54A. Chromatograms are compared for reactions that represent a depletion of InsP_6_ by around 60% of the initial concentration. The major InsP_5_ product in each case is indicated by a *red star*. Mutant K54A shows a predominant 5-hydroxy InsP_5_ peak. Chromatograms of the undigested substrate (InsP_6_ substrate) and an acid hydrolysate of the substrate (InsP*_x_* standards) are shown for reference. The elution volume ranges for the various inositol polyphosphates are highlighted by *vertical colored backgrounds* (note that the notation for the InsP_5_ products is based on the identity of the free hydroxyl group of the intermediate).

### Ligand-driven domain movement along the catalytic cycle

The three-dimensional structures of many HP2P are known. These have been described either in the apo- or product (*i.e.* P_i_)-bound form, or in complex with InsS_6_ (for examples see Table S1). Enzymes for which pairs of apo- and inhibitor complex structures are available do not display discernible domain movements upon ligand binding, including an array of HP2 phytases (11, 12, 37, 41). This is also true for *Bt*MINPP (22). Nevertheless, more localized structural changes have been detected. For example, in *E. coli* AppA, binding of InsS_6_ induces a local rearrangement of residues immediately downstream of the RHG*X*R*X*h catalytic sequence motif (9). Amino acids displaying the largest conformational change were Thr-23 and Lys-24, which “close” the active site cleft over the substrate. This movement allows the side chain of Lys-24 to rotate so as to contact the substrate analog.

To test whether the presence of the steric obstruction presented by the U-loop in *Bl*MINPP might necessitate more profound conformational changes to allow substrate access to and/or product egress from the active site, structures of further representative states along the catalytic pathway were sought. To this end, crystals of the apoenzyme and of an inactive mutant in which the presumed catalytic proton donor Glu-401 was replaced by a glutamine (E401Q) were prepared, the latter in an attempt to trap the catalytic phosphohistidine intermediate. The structures of the apoenzyme and product complex were solved at 1.65 and 1.71 Å resolution, respectively, whereas a data set collected at 2.40 Å resolution from a crystal of the inactive E401Q mutant yielded a structure of the His-45 phosphohistidine derivative.

Analysis of the resulting structures indicates that, unlike previously characterized HP2P, *Bl*MINPP possesses an unusual inherent flexibility (Fig. 4*A*). In the apo-state the enzyme exists in an open conformation with many of the specificity pockets incompletely formed. On binding the substrate analog, the enzyme moves to a closed conformation where the full array of interactions with ligand are present. This movement is not limited to the active center, as is seen in *E. coli* AppA, but rather it propagates to a large region of the α-domain, which we will refer to as the molecular “lid,” which rotates on binding of substrate toward the α*/*β*-*domain. The lid comprises the majority of the α-domain residues, excluding only the residues of helices Ala-222–Ile-231 and Ala-337–Lys-357, which line one side of the active site cleft and the latter of which contributes residues to specificity pocket B. DynDom (42) identified a maximum rotational movement of the lid of 18.1° upon ligand binding, corresponding to 82.3% closure of the moving domain (Fig. 4*B*).

The α*/*β-domain and the remainder of the α*-*domain undergo only limited changes and are considered fixed (RMS deviation 0.55 Å). Lid movements are also seen in the transition from the substrate analog-bound to phosphohistidine intermediate and then to the product-bound form. Unexpectedly, the phosphohistidine intermediate state resembles the open conformation, whereas the product-bound form of the enzyme exhibits a lid rotation of 10° (degree of closure 68.6%) back to a half-closed conformation. Taken together, these snapshots suggest a mechanism whereby the enzyme undergoes a complex structural catalytic cycle during which the lid closes on binding of substrate, then opens fully to expel the first stage product (a lower phosphorylated inositol). Presumably, a second lid motion to a half-closed state then follows to allow hydrolysis of the phosphohistidine intermediate and generation of the bound second stage product (orthophosphate). Finally, lid rotation to regain the open state allows diffusional loss of P_i_ and reattainment of the resting state of the enzyme.

As interdomain screw axes are located in the proximity of bending residues, these amino acids can be considered to be a mechanical hinge with the interdomain screw axis as hinge axis. Two such mechanical hinges were identified by DynDom: the first involving residues clustered on two adjacent loops connecting the α*/*β*-* and α*-*domains, and the second involving two short sequences at the termini of α-helices on the other side of the α*-*domain, away from the bound substrate analog (Fig. 4*B*). Multiple sequence alignments of representative MINPPs and analysis using ConSurf (43) showed high conservation of the residues of the first of these hinges. The two strands of this hinge contain, respectively, the catalytic signature motif RHG*X*R*X*h (beginning at residue 44) and a *GXL*T*X_2_G* motif (beginning at residue 98) also conserved in HP2 enzymes (Figs. 4*C*, Fig. S6).

### Scissile phosphate interaction with Arg-48 is a candidate for initiation of lid closure

Considering its role in substrate positioning, Arg-48 is presumably one of the residues that initiates lid movement, most likely following engagement of a phosphate into the A-specificity pocket of the enzyme (Fig. 4*D*). Docking of a phosphate into pocket A leads to rotation about Arg-48 and propagation of a physical shift to the following polypeptide such that Ser-51 engages with phosphates in specificity pockets C and E, Lys-54 in pockets C and D, and Tyr-55 in pocket C. This region of polypeptide is hydrogen bonded to the second strand in the hinge, whose motion is transmitted to Arg-142, which swings in to engage with phosphates in pockets A and F. Through its interactions with Tyr-53, this motion can be transmitted to the lid subdomain, rotation of which leads to large displacements of residues of the U-loop such that residues Glu-293 and Lys-296 enter specificity pocket D, thus completing coordination of the substrate. This proposal makes ligand-binding the driving force for α-domain movement. In *E. coli* AppA the arginine (Arg-20) corresponding to Arg-48 in *Bl*MINPP has essentially the same role except that movement is propagated only to the residues immediately following it in the sequence. In *Bl*MINPP, however, this small conformational change can be amplified through connections with the U-loop to the lid subdomain with important consequences for interactions with the substrate in specificity pockets C, D, and E.

In the phosphohistidine intermediate, Arg-48 and Arg-142 adopt open-state conformations, neither making contact with the His-45 phospho-group. In fact, this group does not make contact with hinge residues and makes direct contact only with the main chain amide of A400 (A400 forms part of the HAE proton donor motif). It also forms a weaker interaction with Arg-44. The loss of the majority of the interactions observed with the scissile phosphate in the substrate complex are consistent with the relaxation of the phosphohistidine intermediate enzyme to the fully open state. In the phosphate-bound structure, the final stage of the structural catalytic cycle, Arg-48 and Arg-142 swing back to contact the product ion. This ion also makes contact with Arg-44 and Glu-401. Save for a small drift of the ion toward Arg-48, its placement and the interactions it makes with coordinating residues are very similar to that seen for the A-pocket sulfate in the complex with InsS_6_. These interactions require rotation of the lid but can be formed without the necessity for the full extent of lid rotation observed in the closed state. As a consequence, the enzyme exhibits a half-closed state. Presumably, this state will more closely resemble that required for hydrolysis of the phosphohistidine intermediate than the fully open state as alluded to above.

In contrast to the situation for *Bl*MINPP, the structures of the complexes of *Bt*MINPP with InsS_6_ (PDB 4FDU) and with P_i_ (PDB 4FDT) are very similar (RMS deviation 0.24 Å for 333 Cα atoms) (22). Interestingly, these structures most closely resemble the P_i_-bound form of *Bl*MINPP, *i.e.* they adopt a half-closed conformation. No residues in the equivalent of the lid subdomain contribute to specificity pockets in *Bt*MINPP and as a consequence further closure of the α-domain is presumably unnecessary.

### Roles of mobile residues in determining BlMINPP positional specificity

The presence of the U-loop in *Bl*MINPP leads to a range of motions during the catalytic cycle both subtle (for example, rotation of the RHG*X*R*X*h motif to complete coordination of the scissile phosphate in the A-subsite) and marked (most noticeably the 10 Å swing in the U-loop itself leading to residues contacting the bound substrate in specificity pocket D) (Fig. 5*A*). We therefore decided to investigate the roles of specific residues in these regions in determining *Bl*MINPP positional stereospecificity. U-loop residues that on lid rotation approach to within 5 Å of the substrate analog were Glu-293 and Lys-296. No discernible changes in primary hydrolytic positional specificity were observed when these residues were mutated to alanines (Fig. S7). Ser-51 and Lys-54 lie downstream of hinge residues at the domain interface and, on substrate binding move to interact with phosphates in subsites C, D, and E (Fig. 5*A*). An analysis of representative MINPP sequences showed that a serine or a threonine is present at the equivalent of position 51 in at least 80% of cases, with a consensus sequence RHG*X*R*X*L(**S/T**)S*X***K** (residues 51 and 54 in bold) (Fig. S6). In the crystal structure of *Bl*MINPP:InsS_6_, the side chain of Ser-51 interacts with the substrate analog in sites C, D, and E, whereas Lys-54 contacts in sites C and D. Although the mutation S51A leads to an unchanged positional specificity, the K54A mutant shows preference for initial cleavage at the 1D-5-phosphate of phytic acid, rather than for the 1D-4/6-phosphate as displayed by the WT enzyme (Fig. 5*B*). The positional specificity of the K54A mutant resembles closely that observed for the MINPP from *B. thetaiotaomicron*, a predominant 5-phytase (Fig. 1*C*). This is the first experimental observation of an engineered change in positional specificity by a MINPP and suggests that specificity subsites C and/or D may play a role in determining the distribution of lower inositol polyphosphates generated by hydrolysis of InsP_6_ by MINPPs.

## Discussion

In a search for new phytases with elevated thermostability, we have identified a subfamily of predominantly extracellular MINPPs that bear characteristic sequence inserts relative to other HP2P. We name these insertions U-loops. Biochemical, biophysical, and structural characterization of one of these enzymes, the MINPP from *B. longum* subsp. *infantis* ATCC 15697, has provided new perspectives into the roles of this insertion in protein stability and ligand binding. We find that U-loop residues influence thermal stability, recovery of activity after heating, and kinetic parameters for hydrolysis of phytate. Furthermore, this structural feature suggests a basis by which recognition of phytic acid can be extended to specificity subsites underutilized in other HP2P. However, a consequence of the presence of the U-loop is the requirement for large conformational changes during the catalytic cycle. Driven by engagement of a scissile phosphate ion in the A-pocket, the U-loop closes over the active site upon ligand-binding, acting as a “lid extension” of the α-domain, helping to define the enzyme active site and contributing residues to specificity pockets C, D, and E. Although in the closed conformation, the U-loop shields the bound substrate, and presumably hinders diffusional escape of the first stage hydrolysis product. Restoration of the open state conformation in the phosphohistidine intermediate allows product egress, before return to a half-closed state to allow breakdown of the intermediate and generation of the second stage product, orthophosphate.

Phytases, along with other members of the histidine phosphatase superfamily, hydrolyze phosphomonoesters in two distinct steps (21, 44). In the first, the formation of an obligatory phosphohistidine intermediate through nucleophilic attack leads to release of the first stage product, in the case of phytases, a lower phosphorylated inositol. The second step involves recruitment of a water molecule and hydrolysis of the intermediate leading to release of orthophosphate. The structures of HP2 phytases at various stages along this catalytic pathway provide useful insights into the interplay between protein structure and catalytic mechanism. Previous studies have reported details of apo-, substrate analog-, and product-bound forms of various family members but the considerable majority of these have revealed no significant enzyme conformational changes (8, 45). The exception to this is the *E. coli* phytase, AppA, where more pronounced conformational changes occur upon ligand binding, particularly effecting residues 20-25 (Fig. S8) (9). Although Arg-20 in *Ec*AppA moves significantly to form a contact with the scissile phosphate, in other HP2P the corresponding arginine residue is effectively already in a “bound” conformation in the apo-structure. In addition, the main chain of Lys-24 moves by 4.7 Å, leading to a 15 Å shift of the N_z_ atom of its side chain to make a contact with the ligand in the C and D specificity pockets. However, despite the large changes involving this residue, the conformational changes are localized. A more extensive conformational change occurs on ligand binding by *Bl*MINPP. As part of this, the main chain of Lys-54 moves 5.8 Å to adopt its ligand-bound position where it contributes to the same specificity pockets as Lys-24 in *Ec*AppA. Although Lys-24 in *Ec*AppA and Lys-54 in *Bl*MINPP are not aligned in terms of sequence (their α-domains have very different folds) their N_z_ atoms lie only 3.6 Å apart when their InsS_6_-bound structures are superimposed suggesting similar roles. That we have shown Lys-54 to play a role in determining positional specificity in *Bl*MINPP may suggest a similar role for Lys-24 in *Ec*AppA.

Exoenzymes find application in a wide range of biotechnological and industrial processes, and are frequent targets for enzyme discovery (46). Extreme environments are commonly exploited to discover new and robust enzymes well-suited for use in industrial applications. The extracellular MINPPs revealed by phylogenetic analysis in this study are found in a variety of often extreme environments. MINPPs bearing a type A U-loop are typically extracellular membrane-anchored enzymes of Gram-positive bacteria such as *Microbacterium hydrocarbonoxidans* and *B. longum. M. hydrocarbonoxidans* is an actinobacterium adapted to harsh environments. It is able to survive, for example, in oil-contaminated soil (47) or toluene filters (48). Enzymes containing the type B loop are found in a variety of environmental Gram-negative bacteria including *Cupriavidus gracilis,* a heavy-metal resistant bacterium first found in industrial biotopes (49), and *Variovorax paradoxus*, a Gram-negative, β proteobacterium able to utilze a wide array of recalcitrant organic pollutant and heavy metals (50).

The preferred substrate and function of MINPPs *in vivo* are still uncertain, despite there being evidence of a role for these enzymes in a variety of cellular processes and organisms (29–32). *B. longum* subsp. *infantis* ATCC 15697 is a human gut commensal known for its positive role in the early development of the infant gut. It decreases intestinal permeability, displays anti-inflammatory activity in intestinal cells, and has been associated with a lower risk of necrotizing enterocolitis in premature infants (51). The bacterium is genetically well-adapted to coexistence with its human host and it is able to digest human milk oligosaccharides due to the presence of a Human Milk Oligosaccharides gene cluster (HMO cluster I) encoding multiple oligosaccharide transporters and glycosyl hydrolases not found in other bifidobacteria (51). *Bl*MINPP is membrane-anchored and extracellular, and thus exposed to phosphate-containing substrates in human milk, for example, *myo*-inositol polyphosphates (52) or casein phosphopeptides (53). Considering the low and tightly regulated phosphorus levels in human milk compared with other mammals (54), it may be the case that a role for this MINPP is to hydrolyze *myo*-inositol polyphosphates, leading to an increase in the local concentration of bioavailable free phosphate whereas maintaining phosphate homeostasis in the gut (55). However, *Bl*MINPP only hydrolyzes phytic acid to produce *myo*-inositol trisphosphates as an end point (35). This may be related to the unusual population of specificity subsites C, D, and E relative to other MINPPs and HP2P in general.

By far the most common application of microbial HP2P in animal nutrition has been their use as additives to animal feeds (3, 56, 57). Genetically modified phytase crops (58, 59) and transgenic animals (*e.g.* pigs (60)) are other approaches. Multiple inositol polyphosphate phosphatases have yet to find application in any of these areas although chicken MINPP has been suggested as a possible vehicle for the development of transgenic chicken (61). The structural basis for the role of the U-loop in *Bl*MINPP in enhancing protein thermostability and in tailoring the recognition of inositol polyphosphate substrates as described herein will be of use in efforts to alter the specificity and stability of HP2Ps by protein engineering. Indeed, given their prevalence in the environment and inherent catalytic flexibility, MINPPs have the potential to provide a rich source of new enzymes for development for animal feed enzyme applications. Given their lack of positional specificity toward phytate, MINPPs may prove attractive targets for development as a new avenue for feed enzymes, most reasonably utilized in conjunction with highly active but more specific conventional HP2P (62). Certainly, the ability to alter the positional specificity of a MINPP, as demonstrated in this work for *Bl*MINPP, holds promise for the development of highly efficient animal feed enzymes acting synergistically to effect the complete dephosphorylation of phytic acid.

## Experimental procedures

### Phylogenesis

The evolutionary history of clade 2 histidine phosphatases was inferred using the Maximum Likelihood method based on the JTT matrix-based model (63). Amino acid sequences were aligned with MUSCLE (64) and Jalview (65) was used for manual editing of the resulting multiple sequence alignments. Initial trees for the heuristic search were obtained automatically by applying Neighbor-Join and BioNJ algorithms to a matrix of pairwise distances estimated using a JTT model, and then selecting the topology with superior log likelihood value. A discrete Gamma distribution was used to model evolutionary rate differences among sites (4 categories; +G, parameter = 2.1429). The analysis involved 51 amino acid sequences. All positions with less than 5% site coverage were eliminated. That is, fewer than 95% alignment gaps, missing data, and ambiguous bases were allowed at any position. There were a total of 720 positions in the final data set. Evolutionary analyses were conducted in MEGA7 (66).

### Cloning and site-directed mutagenesis

The MINPP gene of *B. longum* subsp. *infantis* ATCC 15697 (*Bl*MINPP; GenBank^TM^ ACJ51391.1) was supplied by Vicente Monedero (IATA-CSIC, Spain). *Bl*MINPP was recloned into the isopropyl thio-β-d-galactopyranoside (IPTG)-inducible pOPINF (pOPIN Vector Suite, Protein Production, UK) and pET28a expression vectors. The sequence was cloned in a truncated form (residues 33-545 of the 623 residue full-length protein) excluding the signal peptide and C-terminal sortase-dependent cell wall-anchoring region, and fused to an N-terminal 3C-protease cleavable His_6_ tag (residues MAHHHHHHSSGLEVLFQ**|**GP, where | indicates the 3C-protease cleavage site; pOPINF vector) or an N-terminal thrombin cleavable His_6_ tag (residues MGSSHHHHHHSSGLVPR**|**GSHMAS, where | again indicates the cleavage site; pET28a vector). All experiments involving BlMINPP with WT sequence utilized purified recombinant protein produced by means of the pOPINF construct, whereas experiments involving site-directed variants used protein generated using the pET28a construct and the modified QuikChange site-directed mutagenesis method reported by Liu and Naismith (67).

### Expression and purification

Transformed Rosetta(DE3) pLysS cell cultures were incubated at 37 °C and 180 rpm. On reaching an OD_600_ of 0.6, induction was performed using 0.5 mm IPTG. Cells were chilled to 25 °C and left to grow overnight. They were then harvested by centrifugation at 4 °C, 5500 rpm for 20 min. Pellets were resuspended in 30 ml of NaH_2_PO_4_ lysis buffer (50 mm NaH_2_PO_4_, pH 7.8, 300 mm NaCl, 200 mm imidazole, 0.5% (v/v) Triton) or Tris lysis buffer (50 mm Tris-HCl, pH 8, 300 mm NaCl, 10 mm imidazole), snap frozen in liquid nitrogen, and stored at −80 °C before thawing and lysis of cells by means of a French Press. The soluble fraction of the sample was separated by centrifugation at 4 °C, 15,000 rpm for 30 min and the overexpressed protein was isolated by Ni-NTA IMAC over a 20-500 mm imidazole gradient at pH.8.0.

pOPINF constructs containing a 3C-protease recognition site were dialyzed overnight in Tris buffer (20 mm Tris-HCl, pH 8.4, 150 mm NaCl, 2.5 mm CaCl_2_) in the presence of a 3C-His-tagged protease at a concentration 40× lower than that of the overexpressed protein. A second Ni-NTA IMAC was carried out for the separation of the 3C-His-tagged protein from the sample. pET28a constructs containing a thrombin recognition site were dialyzed overnight in Tris buffer (50 mm Tris-HCl, pH 7.4, 0.5 m NaCl, 20 mm imidazole). The buffer was replaced and the sample dialyzed again for 2 days in the presence of thrombin at a concentration of 2 units/mg of protein of interest. In both cases, cleaved *Bl*MINPP was concentrated using an Amicon Ultracentrifuge filter unit (10 kDa cutoff) and gel filtered using a HiLoad 16/600 Superdex 75 pg column (GE Healthcare) and a running buffer containing 20 mm HEPES pH 7.4, 150 mm NaCl. Protein samples with a purity of at least 99% as estimated by SDS-PAGE were collected. Estimates of the enzyme concentrations were made from absorbance measurements at 280 nm using a NanoDrop One Microvolume UV Spectrophotometer (ThermoScientific).

### Protein crystallization

Crystals of apoprotein (*Bl*MINPP), of BlMINPP in complex with *myo*-inositol hexakissulfate (*Bl*MINPP:InsS_6_), of the phosphorylated mutant *Bl*MINPP E401Q (*Bl*MINPP-Pi) and *Bl*MINPP E401Q in complex with orthophosphate (*Bl*MINPP:Pi) were obtained by sitting drop vapor diffusion using a protein concentrated to 9 mg/ml in 20 mm HEPES, pH 7.4, 150 mm NaCl storage buffer. For the crystallization of *Bl*MINPP:InsS_6_, co-crystallization plates were set up with the addition of 1 mm InsS_6_ to the protein sample (InsS_6,_ *myo*-inositol hexakissulfate hexapotassium salt, product number ACM28434255, was obtained from Alfa Chemistry, Protheragen Inc.). Drops consisted of 50% protein sample and 50% reservoir solution. *Bl*MINPP apo, *Bl*MINPP:Pi, and *Bl*MINPP:Pi crystals grew in drops with a reservoir solution composed of 0.1 m MES, pH 6.5, 10 mm zinc chloride, 18% (w/v) PEG 6000. Crystals of the *Bl*MINPP:InsS_6_ complex grew in drops with a reservoir solution of 10% (w/v) PEG 1000 and 10% (w/v) PEG 8000. Crystals were harvested, cryo-protected by the addition of 30% (v/v) glycerol or PEG 400 to the mother liquor and frozen in liquid nitrogen.

### X-ray diffraction data collection and crystal structure determination

Diffraction experiments were performed at the beamlines I02 and I03 of the Diamond Light Source (Oxfordshire, UK) using a Pilatus3 6M detector and BART sample changer. Data reduction was performed with xia2 (68). Initial phases for the structure of the *Bl*MINPP:InsS_6_ complex were determined by molecular replacement using the program Phaser (69) and the structure of the complex of *Bt*MINPP with InsS_6_) (PDB ID 4FDU). The phasing of the structure of the *Bl*MINPP:InsS_6_ complex was made difficult by a pronounced conformational change and was achieved by decreasing the resolution cutoff to 4 Å and performing separate molecular replacement searches for each of the *Bl*MINPP domains. Initial phase estimates were used to generate difference maps in Coot (70). Convergence of cycles of rebuilding in Coot and refinement using phenix.refine (71) gave a refined structure for the two copies of the enzyme in the asymmetric unit. Calculation of Polder OMIT maps (38) revealed significant residual electron density in both active sites corresponding to bound InsS_6_. Careful inspection revealed static disorder at both sites and the inhibitor was added to the model in two orientations presenting either the 4- and 6-sulfate bound at the catalytic center. Further refinement yielded a final structural model with *R*_work_ 20.9% and *R*_free_ 23.8% for all data to 1.84 Å resolution. Structure solution and refinement of the apo-, orthophosphate-bound, and phosphohistidine intermediate forms followed by essentially the same methods except that the structure of *Bt*MINPP in complex with orthophosphate (PDB code 4FDT) was used as search model for molecular replacement. All refined structures were validated using MolProbity (72) and the wwPDB Validation Service (RRID:SCR_018135). All data collection and refinement statistics are reported in Table 1.

### Cloning, expression, and purification of BtMINPP and reference HP2 phytases

*Bt*MINPP and the well-studied reference HP2P from *A. niger* (*An*PhyA) and *E. coli* (*Ec*AppA) were cloned and purified as part of this study.

The *An*PhyA gene was codon optimized for expression in *Pichia pastoris* and synthesized by GenScript. For Gateway cloning the gene fragment was amplified with an N-terminal 3C protease site, a C-terminal His_6_ tag, and Gateway recombination adapters by a two-step PCR using the entry vector pDONR207 and the destination vector pPICZα-DEST. The resulting construct was linearized and transformed into *P. pastoris* KM71H (OCH1::G418R) by electroporation and spread onto 6-well LB plates containing 100 μg/ml of kanamycin and 100 μg/ml of zeocin, and incubated at 30 °C for 3-5 days. A single colony was selected and used to inoculate 5 ml of BMGY media (1% yeast extract, 2% peptone, 100 mm potassium phosphate buffer, pH 5.0, 1.34% yeast nitrogen base, 4 × 10^−5%^ biotin, 1% glycerol, 100 μg/ml of kanamycin). After overnight incubation at 30 °C with shaking at 200 rpm, the cells were resuspended in 5 ml of BMMY media (1% yeast extract, 2% peptone, 100 mm potassium phosphate buffer, pH 5.0, 1.34% yeast nitrogen base, 4 × 10^−5%^ biotin, 1% methanol, 100 μg/ml of kanamycin) and incubated for a further 4 days at 30 °C with shaking at 200 rpm. As the recombinant *An*PhyA protein is secreted into the media, supernatants were harvested by centrifugation for 10 min at 4,000 rpm. Prior to purification, the pH of the supernatants was adjusted to pH 8.0 by addition of 10 n NaOH; and precipitate was removed by centrifugation for 10 min at 4,000 rpm. *An*PhyA was subsequently purified from the supernatant by Ni-NTA metal affinity chromatography and stored at −80 °C.

The gene encoding *E. coli* AppA (*Ec*AppA) was amplified from the genome of BL21 (DE3) pLysS and cloned into pOPINB. The constructs was designed for the cytoplasmic IPTG-inducible expression of an N-terminal cleavable His tag protein. The sequence was confirmed by sequencing and the protein expressed in soluble form using the *E. coli* B strain Shuffle Express T7 (73). The protein was then purified by Ni-NTA metal affinity chromatography followed by gel filtration through a HiLoad 16/600 Superdex 75 pg (GE Healthcare) in 200 mm sodium acetate, 150 mm NaCl, pH 4.5, and stored at −80 °C. *Bt*MINPP was expressed and purified according to previously established methods (22).

### Phosphate release assay

This assay allows the determination of the free phosphate released by hydrolysis of InsP_6_ by the molybdenum blue reaction (74). The absorbance of molybdenum blue is measured at 700 nm and is proportional to the P_i_ concentration. A typical calibration curve shows the assay to be in the linear range from 10 μm to 2.5 mm orthophosphate. 5 mm phytic acid dipotassium salt (≥95% pure) was used as substrate. Reactions were performed at room temperature at pH 3.5, 5.5, or 7.4. Reactions of 50 or 100 μl volume were stopped by the addition of equal amounts of a freshly prepared solution made of 4 parts of reagent A (12 mm ammonium molybdate tetrahydrate, 5.4% saturated sulfuric acid) and 1 part of reagent B (0.4 m iron(II) sulfate heptahydrate plus a few drops of saturated sulfuric acid). Absorbance was measured at 700 nm after 30 min using a Hidex Sense plate reader. Control reactions of buffer only, substrate only, and enzyme only were set up simultaneously as well as a calibration curve of increasing concentration of orthophosphate.

### Phytase activity recovery after heating of disulfide mutants

The phytase activity of mutants D1, D2, and D1D2 were assessed and compared with the activity of *Bl*MINPP WT after 30 min incubation at a range of temperatures from 25 to 80°C. After incubation, the samples were cooled to room temperature and mixed with 5 mm InsP_6_ at pH 5.5 and reactions were allowed to proceed for 30 min at room temperature. Phosphate release was measured by means of the Phosphate Release Assay. Results are displayed as micromole of P_i_ per minute per mg of protein.

### Measurement of enzyme kinetic parameters

Reactions of 50 μl were set up in triplicate at fixed concentrations of enzymes (25 nm) and increasing concentration of substrate (50, 100, 200, 400, 600, 800, 1200, and 2500 μm). They were incubated for 5 min at room temperature. The buffer chosen was 200 mm sodium acetate, pH 5.5, 0.15 m NaCl. Reactions were inactivated by addition of molybdenum blue reagent in equal parts and the absorbance at 700 nm was measured after 30 min incubation of the samples with the stopping reagent. Data were processed with the “nls” function provided in R 41 (see https://stat.ethz.ch/R-manual/R-devel/library/stats/html/nls.html), that determines the nonlinear least-squares estimates of the parameters of a nonlinear model. In this analysis, the nonlinear model is the Michaelis-Menten equation. The goodness of fit of the model was confirmed by checking residual error values and *t* test.

### Identification of inositol polyphosphates by HPLC

*myo*-Inositol 1,2,3,4,5,6-hexakisphosphate, dodecasodium salt (InsP_6_, 1 mm, *Zea mays*, Merck, 99% pure, confirmed by HPLC), was used as substrate. Enzymes were used at 25 nm in 200 mm sodium acetate, pH 5.5, 150 mm NaCl. Reactions were stopped at 5 and 10 min by boiling samples at 100 °C for 10 min. Samples were diluted 5× before injection. Inositol polyphosphate standards were generated by the hydrolysis of InsP_6_ in 1 m HCl, 120 °C for 24 h. The HPLC system consisted of a first pump for sample injection (Jasco PU-2089 I Plus–Quaternary inert Pump) connected in series to two CarboPAC PA200 columns (3 × 50 mm, 3 × 250 mm) in which the InsP*_x_* species were efficiently separated (enantiomers, however, cannot be resolved) before reaching a chamber in which they were chaotically mixed with a reagent (0.1% Fe(NO_3_)_2_, 2% HClO_4_), which was injected by a second pump (Jasco PU-1585 Intelligent HPLC Pump). This allows UV absorbance detection at 290 nm (range 1.28 nm, Jasco UV 1575 Intelligent UV-visible detector: 16 μl cell). Samples were separated in a methane sulfonic acid gradient (0- 0.6 m), flow rate 0.4 ml/min, with water as a counter eluent, reagents were injected at a flow rate of 0.2 ml/min. The total run time for each sample was 50 min: 25 min of gradient, 14 min of 0.6 m methane sulfonic acid, 11 min of water. The peak areas were calculated by integration using the software provided by Jasco (ChromNAV, version 1.19.01). The identities of inositol polyphosphates generated during hydrolysis were determined by reference to the retention times of peaks resulting from a standard sample of chemically hydrolyzed InsP_6_ (HCl, 120 °C, 24 h).

### Calorimetry experiments

A VP-DSC (Microcal Inc.) was used for all calorimetry experiments. Initially, 20 buffer readings were taken to build the thermal history of the instrument. The last runs were used as baseline in subsequent data analysis. The temperature gradient was set to 10-110 °C with a scan rate of 200 °C/h, which assured high sensitivity without excessive sharpness of peaks. A pre-scan of 5 min was added at the beginning of each read. 350 μl of sample solution at a concentration of 1 mg/ml was used for each run. Experiments were carried out at pH 3.5, 5.5, and 7.5 (pH 3.5, 0.2 m glycine-HCl; pH 5.5, 0.2 m sodium acetate; pH 7.5, 0.2 m HEPES), representing the typical pH optima observed for MINPP phytase activity (22).

## Data availability

Coordinates and diffraction data for the crystal structures of *B. longum* MINPP in the apo form, in the phosphohistidine intermediate form and in complexes with inositol hexasulfate and phosphate have been deposited in the PDB with accession codes 6RXD, 6RXE, 6RXF and 6RXG.

## Supplementary Material

Supporting Information
