# Supplementary material for: Snapshots during the catalytic cycle of a histidine acid phytase reveal an induced-fit structural mechanism
Source: J Biol Chem. 2020 Oct 14;295(51):17724–37. doi: 10.1074/jbc.RA120.015925 (PMC7762957; doi:10.1074/jbc.RA120.015925)
Supplement: Supporting Information [file supp_295_51_17724__index.html]

Snapshots during the catalytic cycle of a histidine acid phytase reveal an induced fit structural mechanism — Phytase induced-fit structural mechanism — Snapshots during the catalytic cycle of a histidine acid phytase reveal an induced-fit structural mechanism — Phytase Induced-Fit Structural Mechanism — Supporting Information 

# Snapshots during the catalytic cycle of a histidine acid phytase reveal an induced-fit structural mechanism

## Supporting Information

- Supporting Information (to be published online) - Supporting Information
