# Supplementary material for: Snapshots during the catalytic cycle of a histidine acid phytase reveal an induced-fit structural mechanism
Source: J Biol Chem. 2020 Oct 14;295(51):17724–37. doi: 10.1074/jbc.RA120.015925 (PMC7762957; doi:10.1074/jbc.RA120.015925)
Supplement: Supporting Information [file supp_RA120.015925_163335_2_supp_612395_qd7cdg.docx]

**Snapshots during the catalytic cycle of a histidine acid phytase reveal an induced fit structural mechanism**

Isabella M. Acquistapace^1^, Monika A. Ziętek^1^, Arthur W.H. Li^1^, Melissa Salmon^1^, Imke Kühn^2^, Mike R. Bedford^3^, Charles A. Brearley^1^ & Andrew M. Hemmings^1,4*^

^1^ School of Biological Sciences, University of East Anglia, Norwich. U.K.

^2^ AB Vista, Feldbergstrasse, 64293 Darmstadt, Germany.

^3^ AB Vista, Blenheim Road, Marlborough, Wiltshire, U.K.

^4^ School of Chemistry, University of East Anglia, Norwich. U.K.

**Supplementary Information**

**Supplementary Tables**


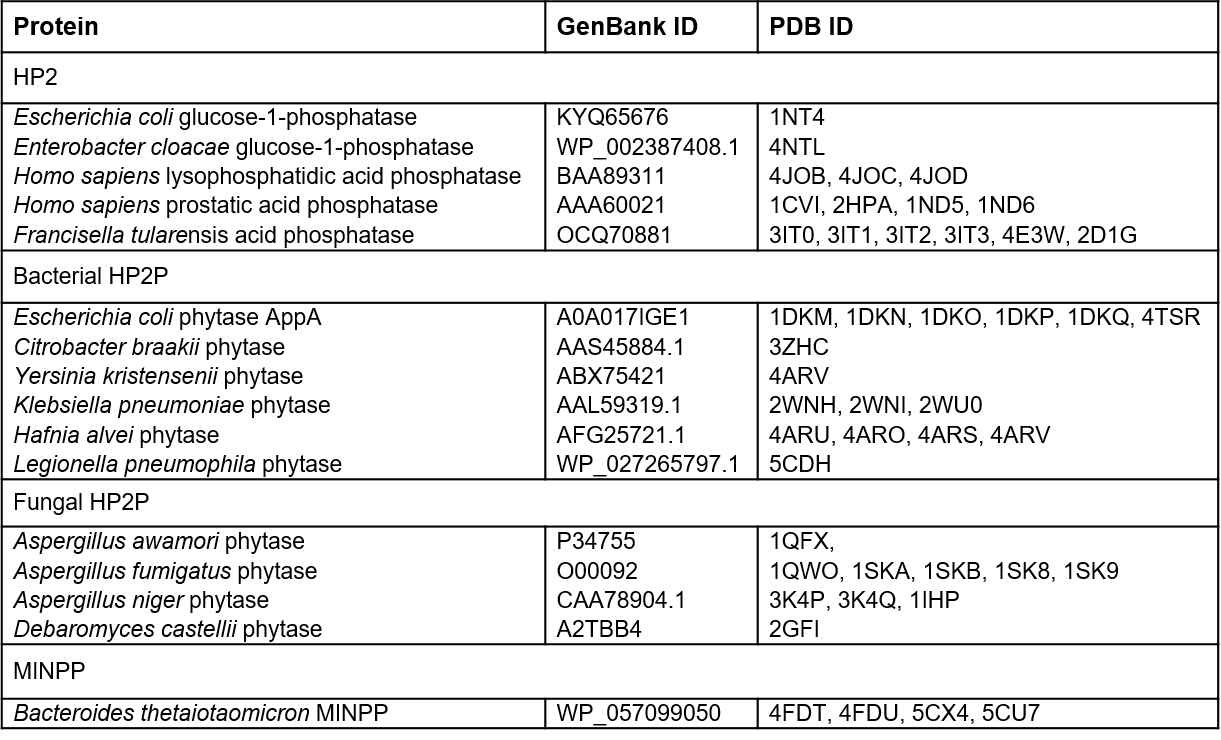


**Table S1. Clade 2 histidine phosphatases of known X-ray crystal structure.** Proteins are grouped as histidine phosphatases which lack phytase activity (HP2), bacterial and fungal HP2 with phytase activity (Bacterial and Fungal HP2P) and multiple inositol phosphate phosphatases (MINPP).


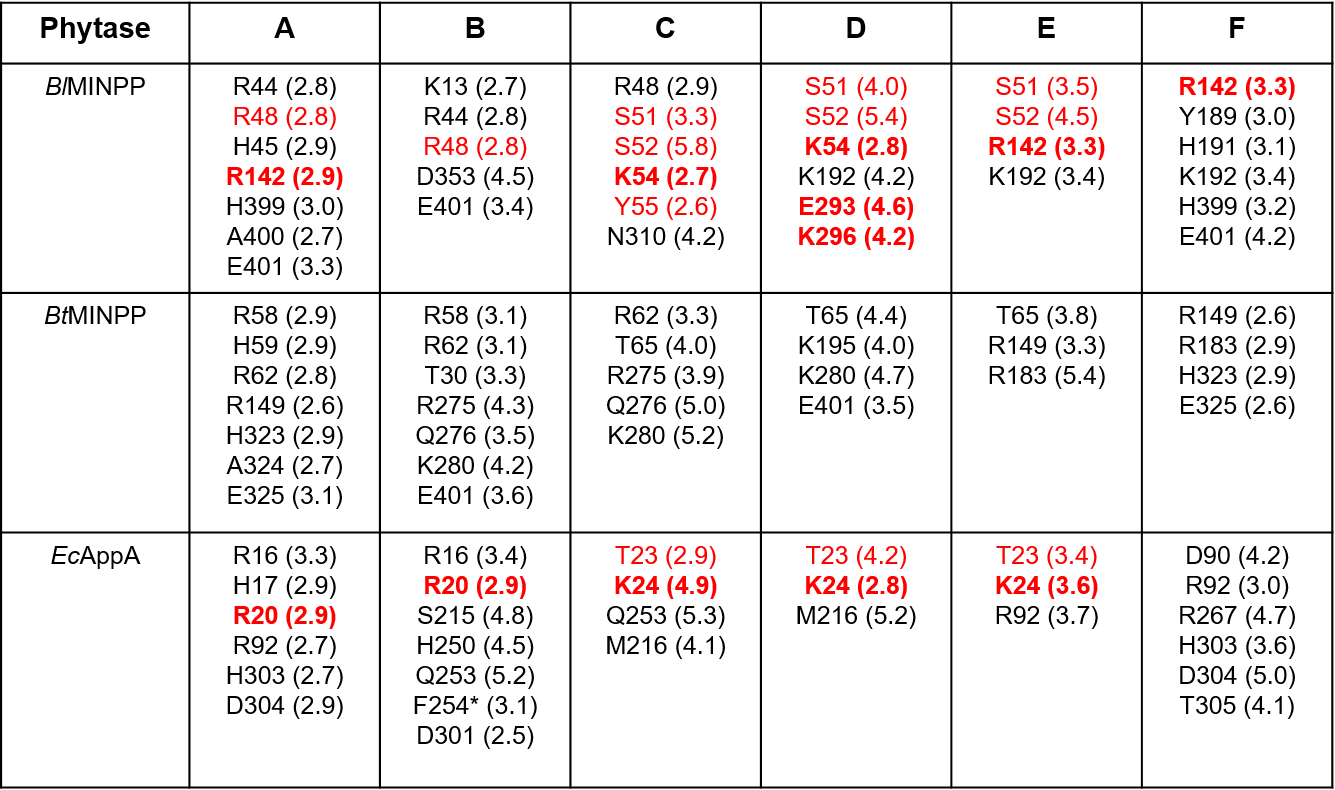


**Table S2.** **Specificity subsite residues of *Bl*MINPP, *Bt*MINPP and *Ec*AppA.** Polar contacts (distance in Å) of active site residues with substrate analogue inhibitor, InsS_6_, in crystal structures of *Bl*MINPP (this study), BtMINPP (PDB entry 4FDU) and EcAppA (PDB entry 1DKQ) arranged according to specificity pocket. Residues in light red move between 2 and 4 Å during ligand binding whilst those in bold red move more than 4Å. Distances based on comparison of PDB entries 4FDT and 4FDU for *Bt*MINPP, and 1DKL and 1DKQ for *Ec*AppA. * indicates a close nonpolar contact.

| **Enzyme** | ***K*_m_ (μM)** | ***k*_cat_ (s^-1^)** | ***k*_cat_/*K*_m_ (s^-1^ μM^-1^)** |
| --- | --- | --- | --- |
| WT | 54.5 ± 8.4 | 46.8 ± 1.2 | 0.9 ± 0.1 |
| S51A | 51.0 ± 4.3 | 44.1 ± 0.6 | 0.9 ± 0.1 |
| E293A | 72.4 ± 12.7 | 79.3 ± 2.7 | 1.1 ± 0.2 |
| K296A | 52.5 ± 4.6 | 32.4 ± 0.5 | 0.6 ± 0.1 |

**Table S3.** **Kinetic parameter values for wild type (WT) *Bl*MINPP and active site mutants.**

**Supplementary Figures**

**List of Figures**

S1. Phytate (D-*myo*-inositol-1,2,3,4,5,6-hexakisphosphate) and the D-numbering system.

S2. Catalytic mechanism of the histidine phosphatase superfamily.

S3. Alignment of amino acid sequences of MINPPs from *Bifidobacterium longum* subsp. *infantis* ATCC 15697 (UniProt B7GTV0), *Bacteroides thetaiotaomicron* (Q89YI8) and the HP2P PhyA from *Aspergillus niger* (P34752).

S4. Single difference (mFo-DFc) omit maps contoured at 3σ.

S5. Kinetic parameters of wild type (wt) and mutant enzymes.

S6. Alignment of amino acid sequences of MINPPs in the region of the first catalytic hinge.

S7. HPLC chromatograms revealing initial products of InsP_6_ hydrolysis by *Bl*MINPP wild type and active site mutants K54A, E293A and K296A.

S8. A comparison of the conformational changes in *Bl*MINPP and *Ec*AppA which occur on ligand binding.


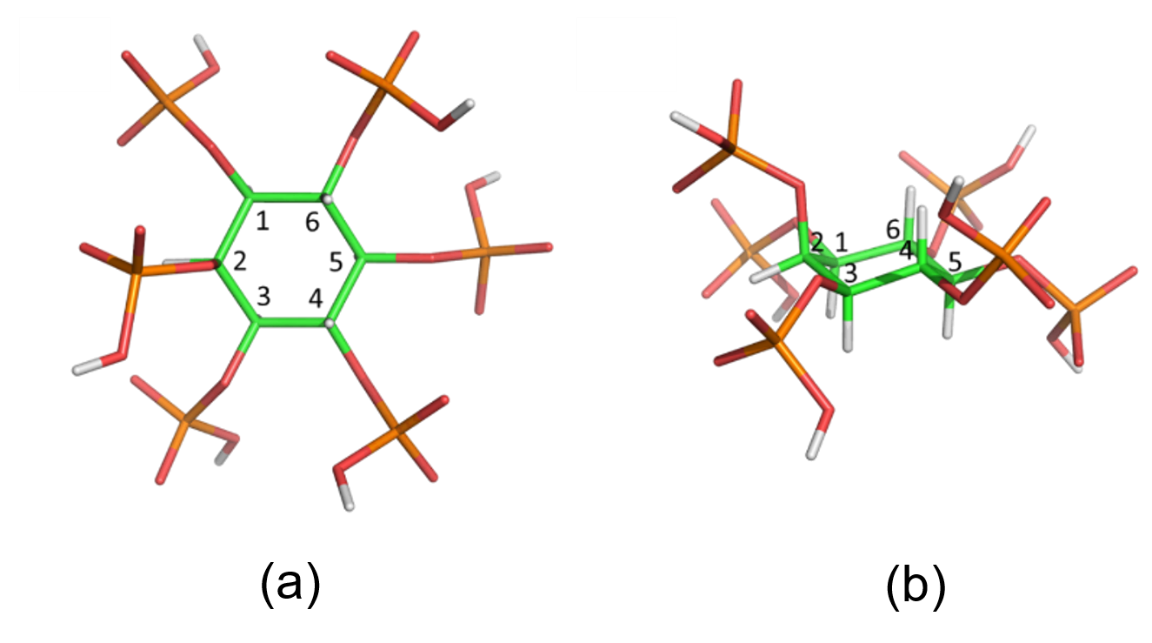


**Figure S1. Phytate (D-*myo*-inositol-1,2,3,4,5,6-hexakisphosphate) and the D-numbering system.** Orthogonal views of a representation of the boat conformer of *myo*-inositol hexakisphosphate observed at physiological pH. (a) top view; (b) side view. Carbon is coloured in green, oxygen in red, phosphorus in orange, hydrogen in white. The phosphate groups are all in the equatorial conformation except that attached to carbon 2 in the inositol ring which is axial. When presented with this axial phosphate oriented towards the viewer, the remaining carbon atoms of the ring are numbered sequentially anticlockwise relative to carbon 2. Note that the order of numbering of the carbon atoms of the inositol ring is reversed in the L-numbering convention.


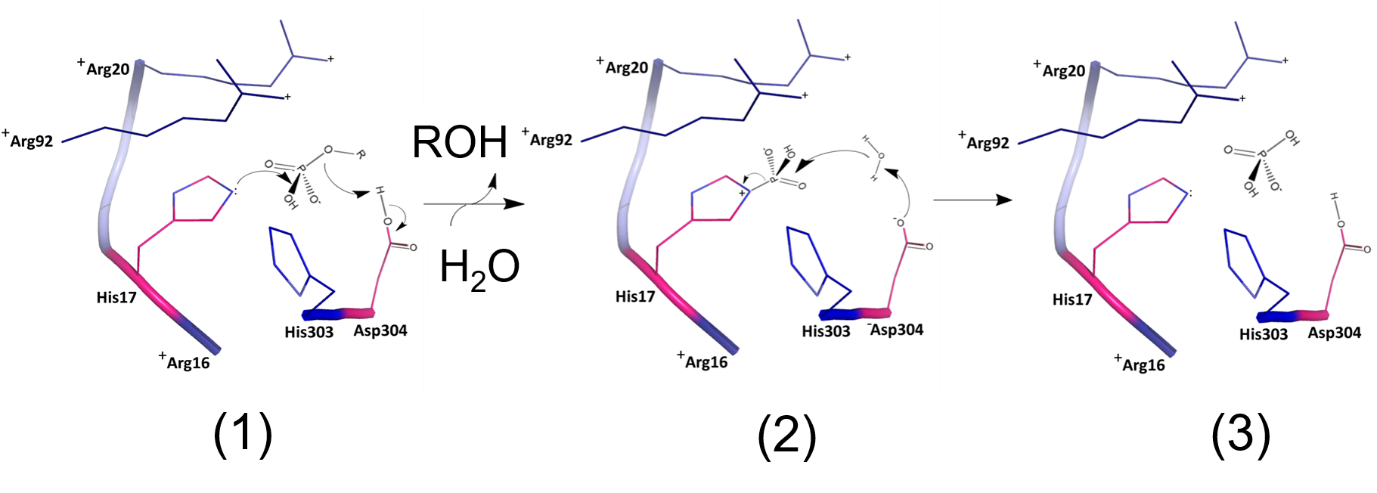


**Figure S2. Catalytic mechanism of the histidine phosphatase superfamily.** Residues numbered as in *E. coli* AppA. Stages shown are (1) Nucleophilic attack. Phosphorylation of the active-site histidine with concomitant donation of a proton by the proton donor (PD) leading to release of the first stage product, IP_5_. (2) Activation of a water molecule and breakdown of the phosphomonoester scissile bond on the phosphohistidine intermediate. (3) Release of the second stage product, orthophosphate. In *Bl*MINPP, the H303-D304 dipeptide is replaced by a H399-A400-E401 tripeptide where the glutamic acid acts as the presumed proton donor.


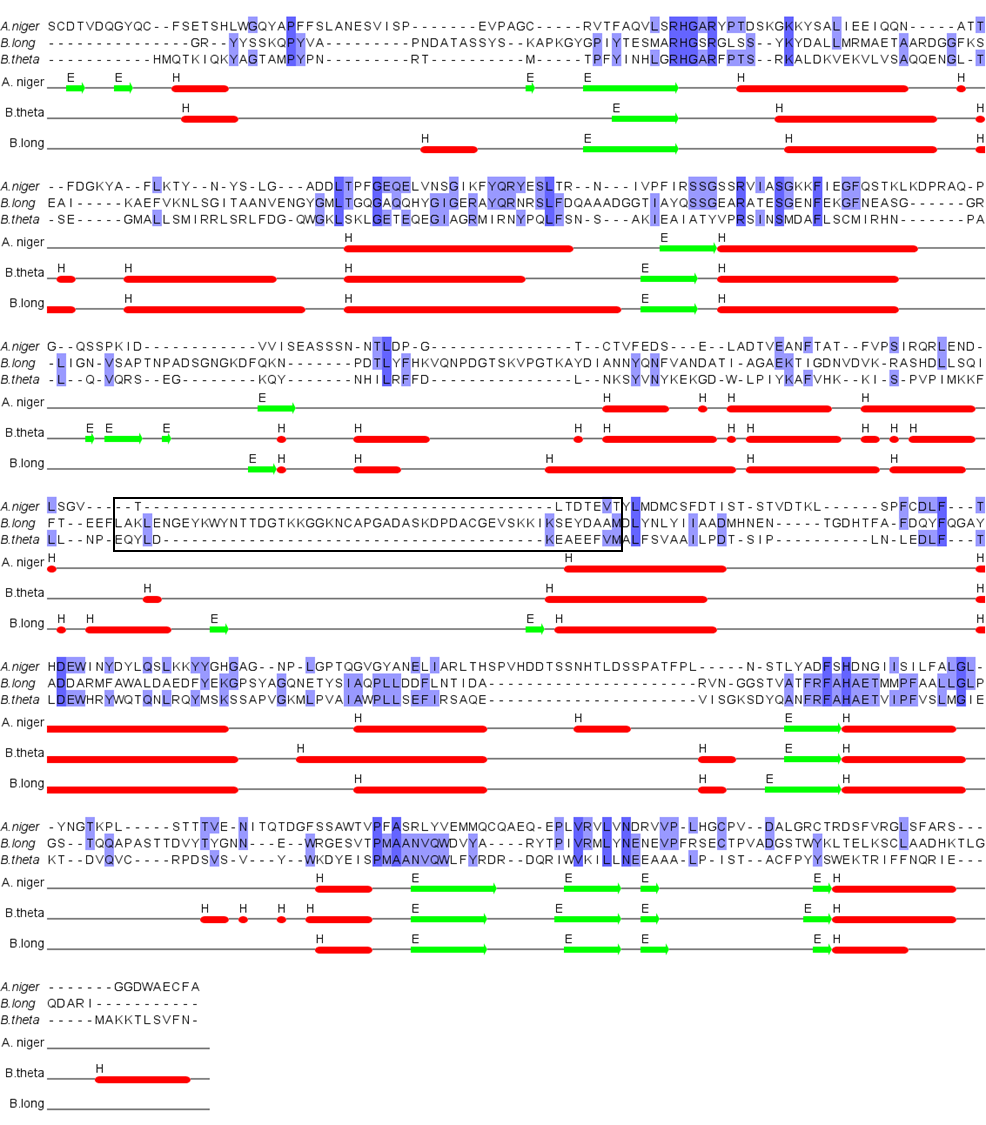


**Figure S3. Alignment of amino acid sequences of MINPPs from *Bifidobacterium longum* subsp. *infantis* ATCC 15697 (UniProt B7GTV0), *Bacteroides thetaiotaomicron* (Q89YI8) and the HP2P PhyA from *Aspergillus niger* (P34752).** Conserved residues shown against a blue background. The position of the U-loop is indicated by a black box. A representation of the proteins secondary structure is reported underneath the multi-sequence alignment (E: strand, H: helix).

**
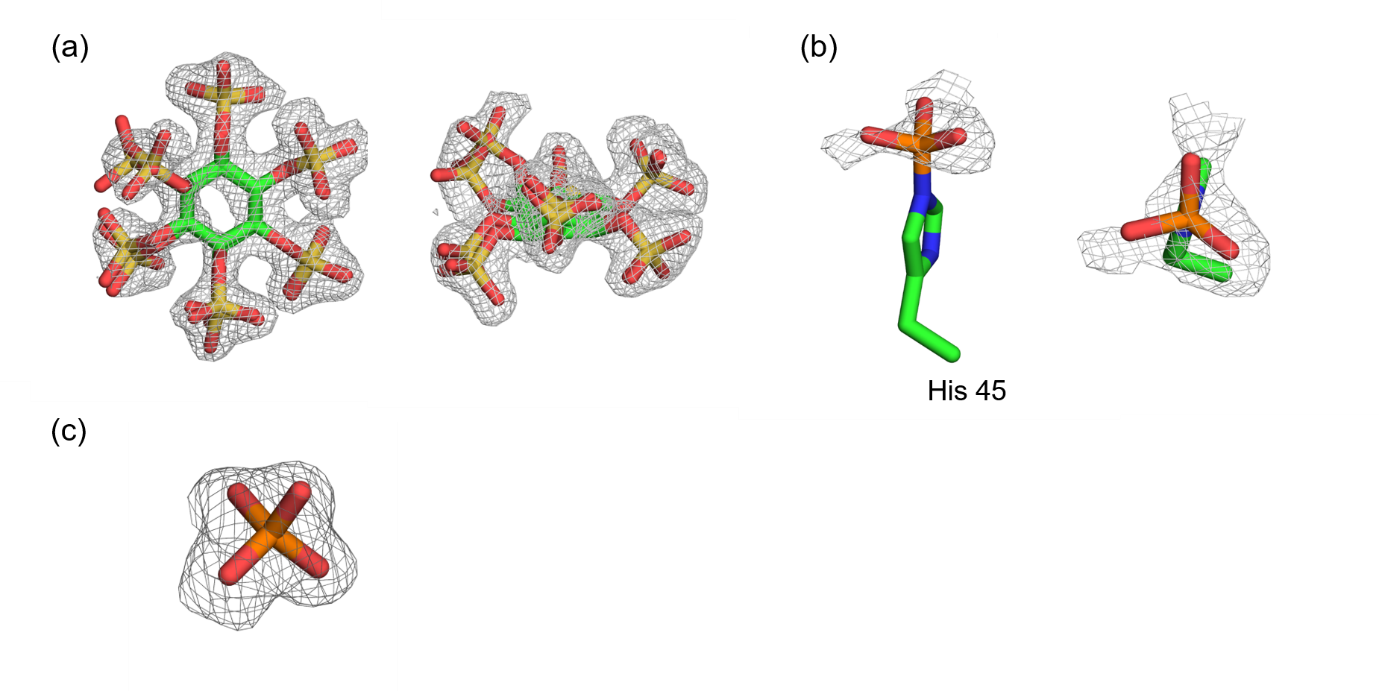
**

**Figure S4. Single difference (mFo-DFc) omit maps contoured at 3σ.** Orthogonal views of **(a)** InsS_6_ (PDB 6RXE) **(b)** Residue His45 (phosphohistidine intermediate) (PDB 6RXF), and single view of **(c)** Orthophosphate complex (PDB 6RXG). In each case, the atoms of the ligand/residue in the final refined structure are overlayed onto the map. Electron density maps shown as grey hatching in each panel.


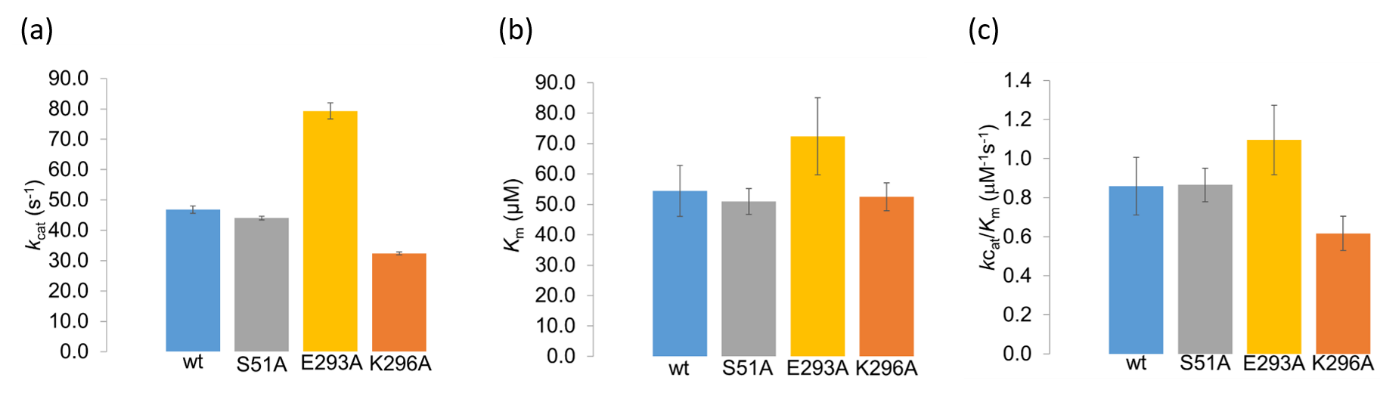


**Figure S5. Kinetic parameters of wild type (wt) and mutant enzymes.** Panels show **(a)** *k*_cat_ **(b)** *K*_m_ and **(c)** *k*_cat_/*K*_m_. U-loop residues mutants (E293A – yellow, K296A – orange) in comparison with the wild-type enzyme (blue). S51, an active site residue which undergoes a large positional shift on substrate binding and contributes to specificity pockets C, D and E is also included (S51A – grey). Error bars show standard deviation of triplicate measurements.


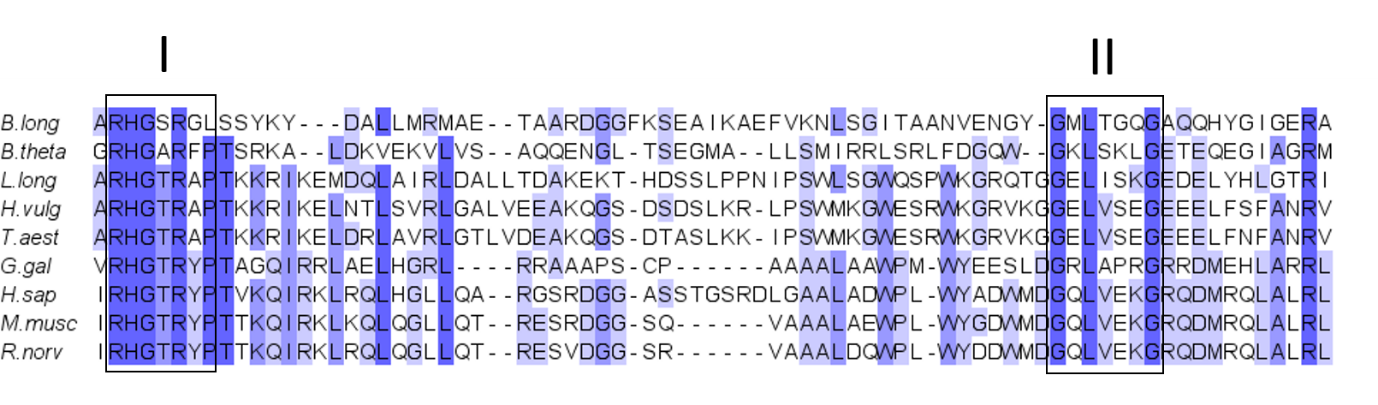


**Figure S6. Alignment of amino acid sequences of MINPPs in the region of the first catalytic hinge.** Sequences taken from *Bifidobacterium longum* subsp. *infantis* ATCC 15697 (UniProt B7GTV0, *Bl*MINPP, *B.long*), *Bacteroides thetaiotaomicron* (Q89YI8, *Bt*MINPP, *B.theta*), Lilium longiflorum (Q0GYS1, *L.long*), *Hordeum vulgare* (A0FHA7, *H.vulg*), *Triticum aestivum* (A0FHB0, *T.aest*), *Gallus gallus* (F1NPQ2, *G.gal*), *Homo sapiens* (Q9UNW1, *H.sap*), *Mus musculus* (Q9Z2L6, *M.musc*) and *Rattus norvegicus* (, *R.norv*). In the sequence of BlMINPP, the catalytic signature motif RHGxRxh begins at residue 44 (box labelled I) and the GxLTx_2_G motif begins at residue 98 (box labelled II). Conserved residues are shown against a coloured background where light blue background indicates residue conservation in at least 40% of sequences, medium blue in 60% and dark blue in 80%.


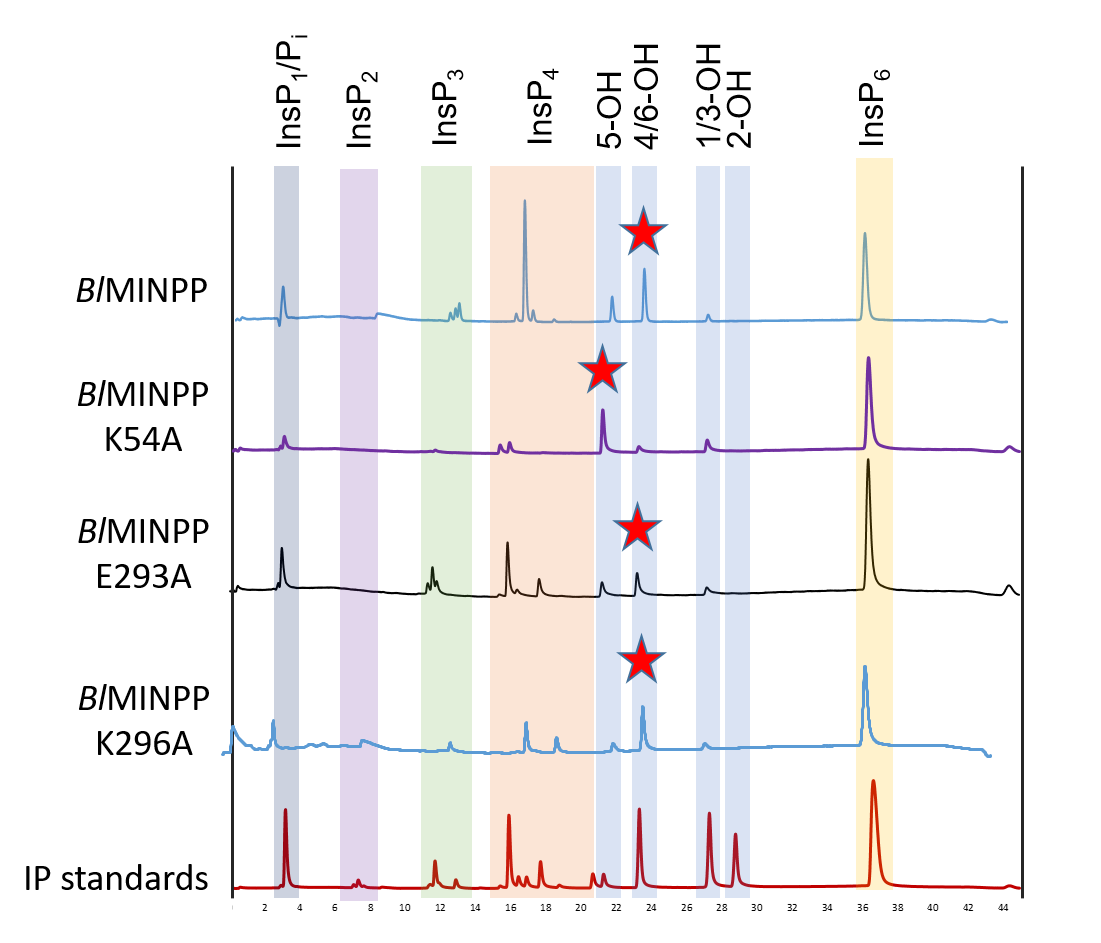


**Figure S7. HPLC chromatograms revealing initial products of InsP_6_ hydrolysis by *Bl*MINPP wild type and active site mutants K54A, E293A and K296A.** Chromatograms are compared for reactions that represent a depletion of InsP_6_ by around 60% of initial concentration. The major InsP_5_ product in each case is indicated by a red star. A chromatogram of an acid hydrolysate of the substrate (InsP_x_ standards) is shown for reference. The elution volume ranges for the various inositol polyphosphates are highlighted by vertical coloured backgrounds (note that the notation for the InsPs products is based on the identity of the free hydroxyl group of the intermediate).


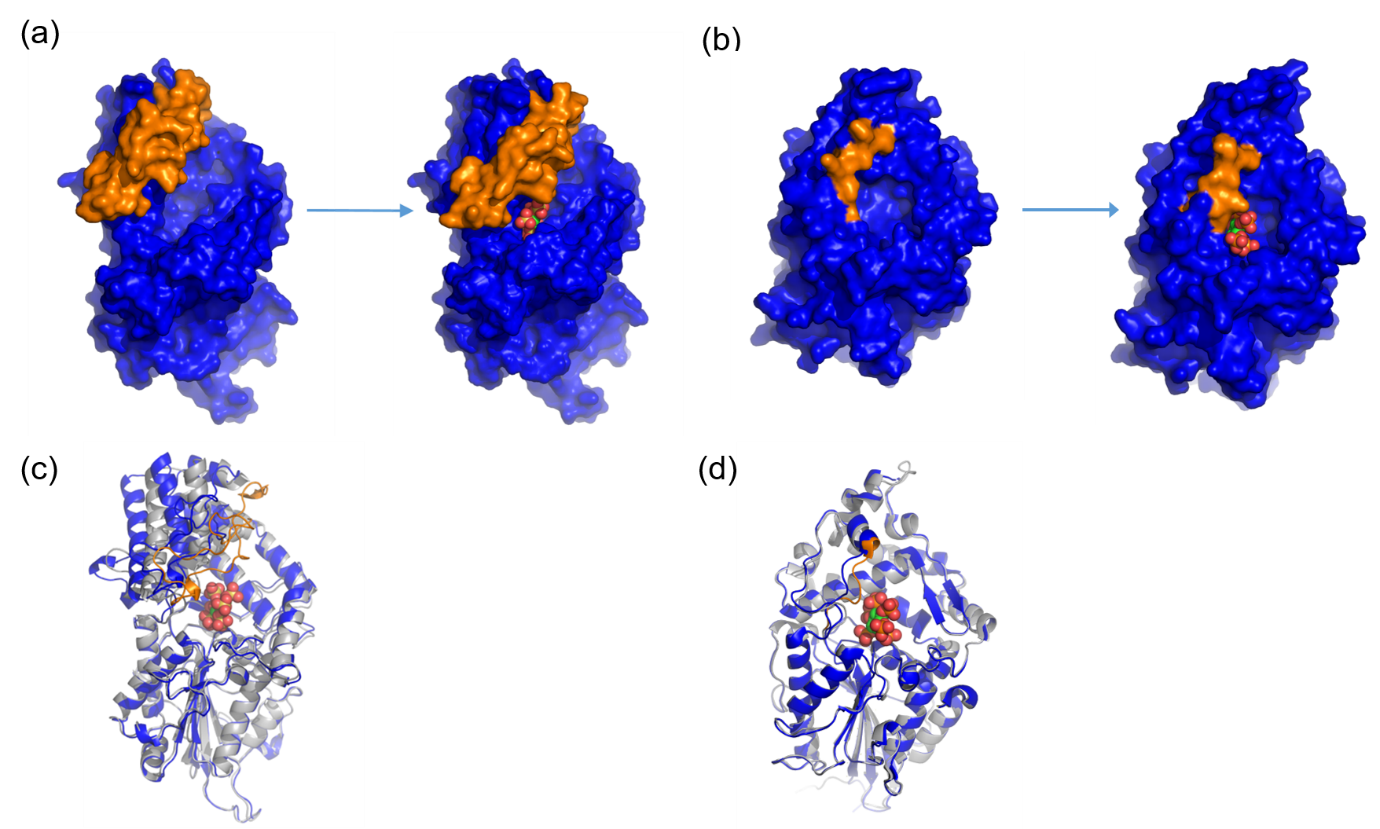


**Figure S8. A comparison of the conformational changes in *Bl*MINPP and *Ec*AppA which occur on ligand binding.** Molecular surface representations of **(a)** *Bl*MINPP and **(b)** *Ec*AppA. For each panel the structure on the left corresponds to the apo-enzyme, and that on the right to its complex with InsS_6_. *Ec*AppA structures taken from PDB entries 1DKL and 1DKQ. For *Bl*MINPP the U-loop residues and for *Ec*AppA the mobile residues (20-28) are coloured orange. Panels **(c)** and **(d)** show the corresponding superpositions of apo- (blue) and InsS_6_-bound (grey) states of *Bl*MINPP and *Ec*AppA, respectively, represented in cartoon format. U-loop residues in *Bl*MINPP and mobile residues (20-28) in *Ec*AppA are coloured orange in the InsS_6_-bound state. InsS_6_ is shown in sphere format and coloured (sulfur-yellow, oxygen-red) in each holo-structure.
